# Safety evaluation of *Aloe vera* soft capsule in acute, subacute toxicity and genotoxicity study

**DOI:** 10.1371/journal.pone.0249356

**Published:** 2021-03-26

**Authors:** Jun Wu, Ying Zhang, Zhongming Lv, Ping Yu, Weiqing Shi

**Affiliations:** Institute of Toxicology and Risk Assessment, Jiangsu Provincial Center for Disease Control and Prevention, Nanjing, Jiangsu, China; Universidade Federal do Rio Grande - FURG, BRAZIL

## Abstract

*Aloe vera* has been widely used in health and nutritional supplements in Chinese herbal medicine. Furthermore, *Aloe vera* production has been an emerging industry for making cosmetics and functional food. However, the reported adverse effects raised questions as to whether *Aloe vera* and its products were safe enough to be used in medicine and health care. In view of this, the safety evaluation of *Aloe vera* products before marketing is very important. The present study aimed to assess the toxicological profile of *Aloe vera* soft capsule (ASC), through acute, subacute toxicity and genotoxicity tests. Male and female ICR mice were received by oral gavage 15000 mg/kg bodyweight of ASC in the acute toxicity test. Male and female SD rats were fed on diet blended with different doses of ASC (equivalent to 832.5, 1665 and 3330 mg/kg bodyweight of ASC) for the subacute toxicity test. In the acute toxicity study, no mortality or behavioral changes were observed, indicating the LD_50_ was higher than 15000 mg/kg bodyweight. In the subacute toxicity test, no significant changes were observed in bodyweight, food consumption, hematological, biochemical or histopathological parameters in the rats exposed. These data suggested that ASC used in this study did not produce any marked subacute toxic effects up to a maximum concentration of 3330 mg/kg bodyweight. In the genotoxicity study, ASC showed no mutagenic activity in the Ames test and no evidence of potential to induce bone marrow micronucleus or testicular chromosome aberrations in ICR mice exposed to 10000 mg/kg bodyweight. Collectively, ASC could be considered safe before it was marketed as a laxative and moistening health food.

## Introduction

*Aloe vera*, one of approximately 420 plant species of Aloe, is a stemless or very short-stemmedperennial succulent or xerophyte. The primary components of *Aloe vera* whole leaf are *Aloe vera* gel and *Aloe vera* latex [1]. The main feature of *Aloe vera* whole leaf is its high water content, ranging from 99.0% to 99.5%. However, the remaining 0.5%-1.0% of the plant leaf contain over 75 potentially bioactive ingredients, such as aloin, aloe emodin, aloe rhein, aloe polysaccharides, etc. [2]. Because of these pharmacologically active ingredients associated with various biological activities, *Aloe vera* has been reported for its antiviral [3], antibacterial [4], anti-inflammatory [5], antimicrobial [6], laxative [7], and anticancer effects [8]. Furthermore, *Aloe vera* production has been an emerging industry for making cosmetics and functional food. *Aloe vera* was also employed as a dietary supplement in a variety of foods and as an ingredient in cosmetic products [9,10].

Although *Aloe vera* has always been considered as a safe health food material [11], sometimes it has not been as safe as generally believed. Questions have been raised as to whether *Aloe vera* and its products are safe to be used in medicine and health-care due to the reported adverse effects [12,13] in humans and genotoxicity, carcinogenicity [14] in both *in vitro* and *in vivo* studies in succession. In 2015, *Aloe vera* whole leaf extract has been classified as a possible human carcinogen (Group 2B) by the International Agency for Research on Cancer [15]. In view of this, the safety evaluation of *Aloe vera* products before marketing is very important, and there is sparse literatures on toxicological safety evaluation on *Aloe vera* products [16,17], such as *Aloe vera* soft capsule (ASC). The current study described an acute toxicity, a battery of genotoxicity studies, and a subacute oral toxicity study, in order to evaluate the toxicological safety of ASC before it was marketed as a laxative and moistening health food.

## Materials and methods

All aspects in this study involving animal use and experimental procedure were performed in compliance with Technical Specifications for Health Food Inspection and Evaluation, published by China National Health Commission in 2003. To achieve a balance between ‘3R’ principle and data adequacy, we followed the National Institutes of Health guide for the care and use of Laboratory animals (NIH Publications No. 8023, revised 1978). All animal use and treatment have been approved by the Office of Laboratory Animal Welfare of Jiangsu Provincial Center for Disease Control and Prevention. Jiangsu Productivity Promotion Center conducts formal review on the work of the office.

### Test substance

*Aloe vera* soft capsule (ASC, Batch No. 20150904) was prepared by the HEALTHMAY biotechnology Co. Ltd in HuBei. The content of ASC was light brown oily semifluid, and consisted of aloin, xylo-oligosaccharide, sunflower seed oil, beeswax. Every 100 g ASC contained 0.32–0.38 g aloin, 45–55 g xylo-oligosaccharide, 44.5–54.5 g sunflower seed oil and 0.15 g beeswax. The recommended daily intake for adults was 2000 mg/day per person. According to the adult weight of 60 kg, the recommended daily intake was 33.3 mg/kg bodyweight.

### Animals, housing conditions and diet

Healthy ICR mice (clean grade) and SD rats (clean grade) were supplied by Shanghai SLAC Laboratory Animal Co. Ltd with the license number SCXK (HU) 2012–0002. ICR mice were used for the acute toxicity study, *in vivo* mouse bone marrow micronucleus assay *and in vivo* mouse testicular chromosome aberration assay. SD rats were used for subacute oral toxicity study. All animals were raised in the barrier experiment. Animals were housed in standard cages with sawdust bedding, with a controlled 12 h light/dark cycle. Temperature was maintained at 20°C to 24°C with a relative humidity of 40% to 70%. Except that SD rats in each dose group were fed with normal diet mixed with different concentrations of ASC, the rest of the animals were provided with standardized diet.

### Acute toxicity study

Mice were divided into 2 groups of 10 animals each (5 males and 5 females), weighing between 18.9 g and 21.6 g. To make a 7500 mg/ml test substance, 15000 mg of ASC was mixed with olive oil, and the final volume was 20 ml. Control group mice received olive oil, and treatment group mice received the 7500 mg/ml test substance, both at the dosage of 15000 mg/kg bodyweight after being food-deprived for 24 h. The volume administrated by oral gavage of each group was 10 ml/kg bodyweight, twice a day, which was equivalent to dose of 15000 mg/kg bodyweight. After the administration of ASC, mice were assessed for signs of toxicity, morbidity and mortality throughout 14 days.

### Genotoxicity studies

#### Ames test

The test strains of auxotrophic Salmonella typhimurium TA97, TA98, TA100 and TA102 (MOLTOX Molecular Toxicology Inc., USA) were employed, in the presence and absence of the metabolism activation system S_9_ (CHI Sientific Inc., USA). Based on the results of a preliminary toxicity dose-range test, five dose levels were set as 8, 40, 200, 1000 and 5000 μg/plate, with three plates for each dose. To make the test substance, 500 mg ASC was mixed with DMSO, and the final volume was 10 ml. The test substance was diluted step by step 5 times to the concentration of each dose group. The solvent control was DMSO and positive controls were standard mutagens as follows, sodium azide (NaN_3_, 1.5 μg/plate) for TA100 without S_9_, 2-aminofluorene (2-AF, 10 μg/plate) for TA97, TA98, and TA100 with S_9_, Dexon (50 μg/plate) for TA97, TA98 and TA102 without S_9_, and 1,8-dihydroxyanthraquinone (1,8-DHAQ, 50 μg/plate) for TA102 with S_9_.

In the test, 0.1 ml of the test substance or control, 0.1 ml of the bacterial suspension and 0.5 ml of either the S_9_ mixture or the phosphate buffer (pH 7.4) were added to top agar. After mixing thoroughly, the mixtures were poured onto minimal glucose agar plates. The plates were incubated for 48 h at 37°C. The number of revertant colonies was counted manually. A positive result was determined where the revertant colony counts were greater than 2-fold those of the solvent control and a clear dose-response relationship was observed.

#### *In vivo* mouse bone marrow micronucleus assay

Fifty ICR mice, weighing 25.3 g-28.9 g, were randomly divided into 5 groups (5 male and 5 female for each group) by bodyweight. Mice were treated by oral gavage (15 ml/kg bodyweight) with either olive oil (negative control) or the test substance at a dose of 2500, 5000, 10000 mg/kg bodyweight. 40 mg cyclophosphamide was dissolved in purified water and diluted to 15 ml as the positive control. The highest dose level was prepared with 10000 mg ASC dissolved in olive oil and the final volume was 15ml. Lower dose levels were prepared through 2-fold serial dilution with olive oil. The negative control group and 3 treatment groups were treated by oral gavage twice, and the interval time of second treatment was precisely 24 h after the first gavage. The positive control group was treated by oral gavage once.

Six hours after the second gavage treatment, mice were sacrificed by cervical dislocation. The sternums were aseptically removed. The spinal canal contents were squeezed out, suspended with calf serum, and smeared onto slides. The slides were fixed with methanol and stained with Giemsa. Red blood cells (RBC) and polychromatic erythrocytes (PCE) were observed under microscopy. The number of PCE was counted from 200 RBC for each animal and PCE/RBC was calculated. For each animal, 1000 PCE were examined to determine the incidence of micronucleus. A positive result of PCE/RBC was determined where PCE/RBC in treatment groups was less than 20% of that in the negative control group.

#### *In vivo* mouse testicular chromosome aberration assay

Sexually mature male ICR mice, weighing 28 g-30 g, were randomly divided into 5 groups. The 5 groups included a negative control group (olive oil), a positive control group (2 mg/kg bodyweight mitomycin), and 3 test substance treatment groups (2500, 5000 and 10000 mg/kg bodyweight). 10000 mg ASC was dissolved in 15 ml olive oil to make the test substance for the high-dose group, which was double diluted with olive oil to make the test substance for the middle-does and low-dose group. 4 mg mitomycin was dissolved in physiological saline to the constant volume of 20 ml. The mice in the positive control group were treated by intraperitoneal injection with volume of 10 ml/kg bodyweight, and other groups were treated by oral gavage with volume of 15 ml/kg bodyweight, once daily for 5 successive days.

Mice were injected 10 ml/kg bodyweight colchicine by intraperitoneal on the ninth day after the last treatment. The mice were sacrificed by cervical dislocation. Bilateral testicles were removed, and adipose tissue were eliminated. The seminiferous tubules were separated gently and fixed by methanol-glacial acetic acid (3:1), then centrifuged at 1000 r/min for 7 min. The suspension was applied on a slide, dried in air, fixed with methanol and stained with Giemsa. 100 metaphase division phase of primary spermatocyte in each mouse were observed under microscopy to determine the incidence of chromosome aberration.

### Subacute oral toxicity study

After acclimating to the laboratory environment for one week, 140 weaning SD rats weighing approximately 79 g to 93 g were assigned randomly to 4 groups (10 males and 10 females in each group). The 4 groups included 1 control group and 3 treatment groups dosed at 832.5, 1665 and 3330 mg/kg bodyweight (equivalent to 25-, 50- and 100-fold of the ASC recommended human dose). Diet for the treatment groups were made by blending the normal diet with 167 g, 333 g and 666 g of ASC, respectively, to the total weight of 20 kg. Rats in the control group were fed on normal diet for 30 days. For each rat, bodyweight, food consumption and survival status were recorded at least twice a week. At the thirtieth day, rats were fasted for 16–18 h with free-drinking water. All rat blood samples were obtained from the abdominal aorta immediately prior to necropsy for hematology and clinical chemistry under pentobarbital anesthesia, then all rats were killed by exsanguinations and were autopsied.

#### Hematology

The whole blood stabilized by the anticoagulant ethylene diamine tetraacetic acid (EDTA) was analyzed using Automatic five-classification blood cell analyzer (ADVIA® 2120, Siemens Corporation). Hemoglobin level (HGB), red blood cell counts (RBC), white blood cells counts (WBC), neutrophils counts (NE), lymphocyte counts (LY), monocyte counts (MO), eosinophil counts (EO) and basophil counts (BA) were evaluated. Clinical chemistry was analyzed with an automatic clinical analyzer (AU640, Olympus Corporation) to determine serum alanine aminotransferase (ALT), aspartate aminotransferase (AST), total protein (TP), albumin (ALB), triglyceride (TG), glucose (GLU), albumin (ALB), blood urea nitrogen (BUN), creatinine (CRE) and cholesterol (CHO).

#### Necropsy and histopathology

The organs such as liver, kidney, spleen, stomach, testes (or ovary) were weighed. Organ-to-bodyweight ratios were calculated as (organ/bodyweight)×100. Organs and tissues were fixed in 10% formalin, embedded in paraffin, stained with hematoxylin and eosin. Histopathological examinations were firstly conducted on the corresponding organs in the high-dose group. If there were pathological changes observed, histological examination were carried out in the mid-dose and low-dose groups.

### Statistical analysis

All data were analyzed by SPSS for windows version 20.0 (SPSS Inc., Chicago, Illinois, USA). In the subacute oral toxicity study, all statistical analyses were stratified by sex and dosage. bodyweight, organ/bodyweight, hematology, and biochemistry data were assessed by ANOVA, incorporating Levene’s test for homogeneity of variance. The means of the treatment groups were compared to the control group using Dunnett’s test or nonparametric methods, according to whether or not that Levene’s test showed equal variances. A two-sided p value of less than 0.05 was considered significant. The data from the *in vivo* mouse micronucleus assay or *in vivo* mouse testicular chromosome aberration assay were analyzed using the χ^2^ test.

## Results

### Acute toxicity study

Orally administered dose of ASC up to 15000 mg/kg bodyweight did not produce any toxicity-induced symptoms and mortality. The LD_50_ of ASC to mice was greater than 15000 mg/kg bodyweight.

### Genotoxicity tests

#### Ames test

The number of revertant colonies in ASC treatment group were not increased greater than 2-fold those of the solvent control in TA97a, TA98, TA100, TA102 strains in the absence or presence of S_9_ (Table 1).

**Table 1:** Bacterial reverse mutation assay conducted with ASC.

Treatment	Dose (μg/plate)	TA97a		TA98		TA100		TA102	
		+S_9_	-S_9_	+S_9_	-S_9_	+S_9_	-S_9_	+S_9_	-S_9_
Untreated control		121±5	101±3	35±1	31±2	135±9	126±8	269±5	252±4
Solvent control		137±4	117±11	36±5	34±3	138±4	129±8	274±73	262±20
ASC	8	131±11	126±8	38±2	37±3	137±3	132±4	269±7	256±9
	40	142±9	138±6	39±3	36±3	136±5	142±12	276±10	277±26
	200	142±5	134±15	39±3	34±2	125±4	123±13	264±7	255±8
	1000	140±20	132±15	38±3	36±1	151±12	128±6	274±7	259±6
	5000	175±5	122±11	36±5	32±4	144±6	137±5	275±10	252±7
Dexon	50.0		1190±98*		1075±232*				1079±197*
NaN_3_	1.5						1171±40*		
2-AF	10.0	1001±250*		848±73*		1002±16*			
1,8-DHAQ	50.0							1075±103*	

Data are shown as Mean ± SD revertants/plate for three replicates for each concentration in each experiment.

* Statistically significant difference from control values (p<0.05).

#### *In vivo* mouse bone marrow micronucleus assay

The micronucleus frequency in the positive control groups was significantly higher than that in each ASC treatment group and the negative control group (p<0.05). The PCE/RBC in each ASC treatment group was not less than 20% of that in the negative control group (Table 2).

**Table 2:** The results of the *In vivo* mouse bone marrow micronucleus assay.

Sex	Dose (mg/kg bodyweight)	PCE/RBC	Micronucleus counts (counts/5000PCE)	Micronucleus frequency (‰)
Female	0	1.01±0.07	8	1.60±0.55
	2500	0.97±0.03	7	1.40±0.55
	5000	0.98±0.04	8	1.60±0.89
	10000	1.01±0.05	7	1.40±0.55
	Cyclophosphamide^a^	0.93±0.05	120	24.00±3.39*
Male	0	1.02±0.07	8	1.60±1.14
	2500	1.00±0.04	8	1.60±0.89
	5000	1.03±0.03	7	1.40±1.14
	10000	1.03±0.07	7	1.40±0.55
	Cyclophosphamide^a^	0.98±0.10	100	20.00±3.16*

*Statistically significant difference from control values (p<0.05).

^a^ Positive control.

***In vivo* mouse testicular chromosome aberration assay.** The differences in chromosome aberration frequency between each ASC treatment group and the negative control group showed no statistical significance (p>0.05), indicating that ASC had no mutagenicity in mice (Table 3).

**Table 3:** The results of *In vivo* mouse testicular chromosome aberration assay.

Dose	aberration counts	aberration type		chromosome aberration frequency (%)
		fragment	chain quadrivalent	
0	2	2	0	0.4±0.5
2500	2	2	0	0.4±0.5
5000	1	1	0	0.2±0.4
10000	1	1	0	0.2±0.4
Mitomycin^a^	43	42	1	8.6±1.5*

*Statistically significant difference from control values (p<0.05).

^a^ Positive control.

### Subacute oral toxicity study

All rats in each treatment group showed no significant difference in bodyweight, food intake and food utilization rate (7, 14, 21, 30-day and total) compared with the control group (p>0.05) (Tables 4–6).

**Table 4:** Effects on bodyweight gain of rats treated orally with ASC for 30 days.

Sex	Dose (mg/kg bodyweight)	Initial Weight (g)	7-day (g)	14-day (g)	21-day (g)	30-day (g)	Total (g)
Female	0	83±4	129±8	172±10	195±15	223±18	140±18
	832.5	82±3	130±7	169±10	192±13	222±17	140±14
	1665	82±4	125±9	164±10	185±14	212±20	130±17
	3330	81±4	121±9	161±15	183±16	209±19	128±16
Male	0	86±4	149±8	218±12	277±15	338±19	251±17
	832.5	87±5	151±11	219±17	278±20	335±29	248±25
	1665	87±3	146±6	214±8	270±11	334±13	248±13
	3330	86±4	144±9	208±12	266±11	326±11	240±12

Data are shown as the Mean ± SD (n = 10).

**Table 5:** Effects on food consumption of rats treated orally with ASC for 30 days.

Sex	Dose (mg/kg bodyweight)	7-day (g)	14-day (g)	21-day (g)	30-day (g)	Total (g)
Female	0	101±8	136±6	140±11	139±16	516±31
	832.5	103±11	130±17	135±16	142±21	510±60
	1665	104±10	135±15	134±16	135±11	508±30
	3330	98±11	129±15	129±14	140±16	495±25
Male	0	119±10	170±10	194±11	194±12	676±37
	832.5	119±10	158±18	192±15	187±18	656±56
	1665	118±6	164±11	195±14	193±15	669±43
	3330	113±9	162±15	193±9	187±9	655±27

Data are shown as the Mean ± SD (n = 10).

**Table 6:** Effects on food utilization rate of rats treated orally with ASC for 30 days.

Sex	Dose (mg/kg bodyweight)	7-day (%)	14-day (%)	21-day (%)	30-day (%)	Total (%)
Female	0	44.8±6.4	32.1±7.6	16.1±4.6	20.4±3.9	27.1±2.7
	832.5	46.9±4.4	30.2±3.9	17.2±3.7	20.6±4.4	27.4±1.8
	1665	41.0±5.5	29.0±4.3	15.8±5.0	20.7±8.9	25.5±3.0
	3330	41.0±3.2	30.9±5.7	17.4±2.8	19.0±5.2	25.9±2.5
Male	0	52.8±3.5	40.4±3.4	30.4±1.6	31.5±3.3	37.2±1.6
	832.5	53.3±4.7	43.4±5.0	30.6±2.7	30.7±3.4	37.8±1.7
	1665	50.9±2.7	41.3±3.3	28.5±3.7	33.6±5.2	37.0±1.9
	3330	51.9±4.7	39.4±3.9	29.8±5.4	32.0±3.7	36.6±1.6

Data are shown as the Mean ± SD (n = 10).

EO levels in male rats of the 3330 mg/kg bodyweight dose group and BA levels in female rats of the 832.5 mg/kg bodyweight dose group were significantly lower than those in the control group (p<0.05). AST and TG levels in male rats of the 832.5 mg/kg bodyweight dose group were significantly lower than those in the control group (p<0.05) (Tables 7 and 8).

**Table 7:** Effects on hematological parameters in rats treated orally with ASC for 30 days.

Sex	Dose (mg/kg bodyweight)	WBC (10^9^/L)	RBC (10^12^/L)	HGB (g/dL)	NE (%)	LY (%)	MO (%)	EO (%)	BA (%)
Female	0	6.0±1.9	7.0±0.4	12.6±0.5	14.0±4.0	79.6±5.1	3.0±0.8	2.3±0.6	0.12±0.06
	832.5	5.8±1.7	7.1±0.3	13.3±0.6	13.8±5.4	80.2±5.7	2.9±0.5	2.0±0.8	0.20±0.08*
	1665	6.5±2.2	7.2±0.4	12.9±0.5	13.3±4.0	81.5±4.6	2.5±0.8	1.8±0.6	0.13±0.05
	3330	6.2±1.5	7.2±0.5	12.9±0.7	15.2±6.2	79.6±7.1	2.7±0.8	1.6±0.4	0.15±0.05
Male	0	8.7±1.4	6.9±0.3	12.7±0.7	15.1±7.1	80.3±7.7	2.3±0.9	1.4±0.5	0.17±0.07
	832.5	8.9±1.4	7.0±0.5	12.8±0.9	12.0±1.5	83.7±1.7	2.4±0.5	1.1±0.2	0.19±0.06
	1665	8.3±1.2	6.9±0.2	12.6±0.4	14.5±6.9	80.6±7.6	2.7±1.0	1.4±0.5	0.20±0.07
	3330	8.6±1.7	6.9±0.4	12.7±0.7	14.6±3.3	81.5±3.6	2.3±0.5	1.0±0.3*	0.16±0.07

Data are shown as the Mean ± SD (n = 10).

* Statistically significant difference from control values (p<0.05).

**Table 8:** Effects on clinical chemistry parameters in rats treated orally with ASC for 30 days.

Sex	Dose (mg/kg bodyweight)	ALT (U/L)	AST (U/L)	GLU (mmol/L)	TG (mmol/L)	CHO (mmol/L)	TP (g/L)	ALB (g/L)	BUN (mmol/L)	CRE (μmol/L)
Female	0	36±6	105±15	6.3±0.4	0.98±0.25	2.71±0.48	64±6	33.5±3.5	4.8±0.6	26±3
	832.5	36±51	110±13	5.9±0.4	1.05±0.42	2.42±0.25	64±4	33.7±2.4	5.1±0.5	27±2
	1665	38±7	113±13	6.1±0.5	0.80±0.25	2.46±0.36	61±3	32.1±1.7	5.1±0.9	26±2
	3330	39±8	125±39	6.2±0.4	0.86±0.38	2.36 ±0.34	61±3	31.9±1.9	4.7±0.7	25±3
Male	0	40±4	113±13	6.3±0.7	0.76±0.29	2.06±0.22	57±1	28.4±1.2	4.8±0.9	25±2
	832.5	44±6	133±25*	6.4±0.7	1.20±0.55*	2.05±0.22	57±2	28.5±1.3	5.0±0.7	23±3
	1665	39±8	112±10	6.1±0.7	0.97±0.47	2.16±0.21	56±2	28.2±0.5	4.4±0.7	23±1
	3330	43±5	122±15	5.9±0.6	0.99±0.45	2.21±0.34	57±2	28.8±0.8	4.7±0.6	24±1

Data are shown as the Mean ± SD (n = 10).

* Statistically significant difference from control values (p<0.05).

Compared with the control group, there were no significant differences in bodyweight before sacrifice, liver, kidney, spleen and testis weight as well as corresponding organ to bodyweight ratio among treatment groups (p>0.05) (Tables 9 and 10).

**Table 9:** Effects on organ weights of rats treated with ASC for 30 days.

Sex	Dose (mg/kg bodyweight)	bodyweight before sacrifice	Liver (g)	Kidney (g)	Spleen (g)	Testis (g)
Female	0	216±17	7.93±0.85	1.79±0.15	0.50±0.06	-
	832.5	211±15	8.00±0.91	1.67±0.15	0.52±0.06	-
	1665	206±20	7.50±0.92	1.68±0.20	0.50±0.09	-
	3330	200±19	7.15±1.20	1.67±0.16	0.45±0.09	-
Male	0	321±17	11.15±0.87	2.54±0.27	0.76±0.08	3.13±0.21
	832.5	321±25	11.25±1.35	2.60±0.23	0.80±0.15	3.27±0.16
	1665	319±12	11.39±0.59	2.56±0.25	0.84±0.10	3.26±0.17
	3330	310±11	10.50±0.96	2.42±0.19	0.79±0.10	3.21±0.22

Data are shown as the Mean ± SD (n = 10).

**Table 10:** Effects on organ to bodyweight ratio (g/100 g bodyweight) of rats with ASC for 30 days.

Sex	Dose (mg/kg bodyweight)	Liver (g/100 g)	Kidney (g/100 g)	Spleen (g/100 g)	Testis (g/100 g)
Female	0	3.68±0.30	0.83±0.06	0.23±0.03	-
	832.5	3.77±0.21	0.79±0.03	0.25±0.02	-
	1665	3.63±0.21	0.82±0.09	0.24±0.05	-
	3330	3.57±0.38	0.84±0.06	0.23±0.03	-
Male	0	3.47±0.16	0.79±0.07	0.24±0.02	0.98±0.06
	832.5	3.50±0.20	0.81±0.07	0.25±0.04	1.02±0.07
	1665	3.56±0.12	0.80±0.06	0.26±0.03	1.02±0.05
	3330	3.38±0.25	0.78±0.07	0.26±0.03	1.03±0.07

Data are shown as the Mean ± SD (n = 10).

The results of histopathological examinations showed 1 female rat in the control group had mild hyperemia of spleen red pulp, and 1 male rat in high dose group had hepatic lobular cell lipid change. in addition, no abnormal changes were observed in liver, kidney, spleen, stomach, testis and ovary tissues.

## Discussion

Aloin was the main functional component of ASC. Aloin, a bioactive ingredient extracted from *Aloe vera* has been indicated to induce anti-inflammatory [18], antioxidant [19] and antitumour effects [20]. In addition, xylo-oligosaccharide (XOS), another component in ASC has been proved to demonstrate excellent physicochemical and physiological properties, including antioxidant activity [21], antimicrobial activity [22], immunomodulatory action [23]. However, the safety of the *Aloe vera* product composed of aloin and XOS was still unknown. Thus we have investigated the acute, subacute toxicity and genotoxicity of ASC, aiming to figure out some toxicological changes induced by ASC.

We found the bodyweight gain and food consumption in the 3300 mg/kg group were fewer than that in the 0 and 832.5 mg/kg groups, although the differences were not statistically significant. In recent decades, *Aloe vera* has been reported to be able to cure constipation, help flush out toxins and wastes from the body and promote digestion [24], which may be the reason for the decrease of the bodyweight gain and food consumption in the 3300 mg/kg group. Meanwhile, reduced food consumption could also lead to the loss of the bodyweight gain. It was similar with the outcomes from some long term studies, which reported a significant bodyweight loss during their studies [25,26]. In our studies, no statistically significant changes in the bodyweight changes may be due to the relatively shorter duration of diarrhoea.

Some studies have indicated that *Aloe vera* downregulated inflammatory cytokines, which played an important role in inflammatory cells genesis [27,28]. Furthermore, it was reported that *Aloe vera* whole leaf freeze-dried powder might induce the reduction in WBC and LY [29]. In this study, the WBC and LY in the 3300 mg/kg group were not fewer than the control group. Hematology results showed that some statistically significant changes were sporadically appeared in the study. However, no dose-dependent relationship could be associated with these changes, and it was hard to ascertain that ASC induced these changes. The results were generally consistent with the outcomes of a Wistar rat 2-year study [26].

Upon analysing the ratios, we found that organ-to-bodyweight ratio of livers was decreased in the 3300 mg/kg group, compared with that in the control group, and there was decreased in the absolute organ weight of livers as well. Besides, no statistically significant differences in organ weights and ratios were recorded. *In vitro* studies showed that aloe emodin exhibited cytotoxicity in human liver HL-7702 cells [30] and HepaRG cells [31]. Conversely, another *in vivo* study has indicated *Aloe vera* can attenuate acetaminophen-induced hepatitis [32]. Histopathologically, hepatic lobular cell lipid could be noted in 1/10 male rats in the 3300 mg/kg group. Nevertheness, no statistically significant differences in relevant serum biochemistry results can be found. Therefore, it was hard to determine the possibility of some inner relationship between ASC and hepatic lobular cell lipid changes.

Muller and colleagues investigated the genotoxicity of aloe emodin using micronucleus test [33]. At micromolar concentrations, aloe emodin induced concentration-dependent increases in micronuclei in L5178Y cells. The results of safety studies on a high-purity *Aloe vera* gel demonstrated that the gel was nonmutagenic in the Ames test, the bone marrow micronucleus assay and the chromosomal aberration test [34]. The results of the present series of genotoxicity studies on ASC showed no evidence of mutagenic or clastogenic effects in either somatic or germ cells. The Ames test was negative in all tester strains to the limit dose of 5000 μg/plate. There was no indication of micronucleus induction in the bone marrow cells of mice treated at up to 10000 mg/kg bodyweight dose level, and there were no effects on the incidence of chromosome aberration in mice treated at up to 10000 mg/kg bodyweight dose level.

## Conclusions

In brief, these data suggested that ASC did not produce any marked subacute toxic effects up to a maximum concentration of 3330 mg/kg bodyweight. In addition, the results of the acute toxicity test and genotoxicity test were also negative. These data provided supportive evidence for the safety of ASC that may be used as a laxative and moistening health food.

## Supporting information

S1 File Acute toxicity. (PDF)

S2 File Ames test. (PDF)

S3 File In vivo mouse bone marrow micronucleus assay. (PDF)

S4 File In vivo mouse testicular chromosome aberration assay. (PDF)

S5 File Subacute oral toxicity-bodyweight. (PDF)

S6 File Subacute oral toxicity-organ weight. (PDF)
