# Supplementary material for: Safety evaluation of Aloe vera soft capsule in acute, subacute toxicity and genotoxicity study
Source: PLoS One. 2021 Mar 26;16(3):e0249356. doi: 10.1371/journal.pone.0249356 (PMC7997006; doi:10.1371/journal.pone.0249356)
Supplement: S1 File — (PDF) [file pone.0249356.s001.pdf]

急性经口毒性试验原始记录

样品编号：(毒) GZ02020160028　　试验日期：2016 年 10 月 12 日 —2016 年 10 月 27 日

动物：☐____ 大鼠 ☑ ICR 小鼠，♀ 10 只，♂ 10 只，动物购自 上海斯莱克实验动物有限责任公司 购买证号：2015000524400

动物试验环境：1009 室，体重秤 05- 718 ，温度 20 — 24℃，　相对湿度 40 — 70%

一、动物进入实验室后适应性饲养 3 d，试验前 ☑禁食过夜 ☐禁食____h，不禁水；

二、试验方法：☐Horn 氏法　☐限量法　☑其它：最大耐受量试验法

三、动物体重（g）及分组：按照动物随机分组操作规程进行随机分组。

编号	1	2	3	4	5	6	7	8	9	10	12	13	14	15	16	17	18	19	23	24	$\bar{x} \pm s$
♀ 组别																					
0w	20.5	19.9	18.9	19.7	20.0	19.0	19.4	~~18.6~~ 19.6 吴俊	19.3	18.9											19.5±0.5
1w	25.4	24.5	23.1	24.5	24.4	23.2	24.3	24.3	23.5	23.5											24.1± 0.7
2w	29.3	28.2	26.2	28.3	28.7	26.8	29.0	28.4	27.2	27.7											28.0±1.0
♂ 组别																					
0w	19.9	21.6	20.8	21.0	19.7	20.5	20.5	20.3	21.5	19.0											20.5±0.8
1w	26.4	28.4	27.7	28.4	26.4	27.8	27.4	28.2	29.1	25.9											27.6±1.0
2w	32.0	34.2	33.0	34.7	32.3	33.6	33.5	34.6	35.2	31.7											33.5 ± 1.2

四、样品配制及给药方法：电子天平 05- 268

样品前处理：☑无 ☐有：

剂量组 (mg/kg b.wt.)	称样品量（mg）	溶剂	溶剂加至（ml）	用量 (ml/kg b.wt)
15000	15000	色拉油	20	~~20~~ 10 吴俊 [illegible]灌胃

2 次灌胃给予，☑间隔时间 4 h，染毒后 4 h 给食。观察期 14 d，观察并详细记录动物的中毒表现、死亡数和死亡时间。中毒死亡和人道处死的动物，进行大体解剖检查。

☐其他：

五、结果：

剂量组 (mg/kg b.wt.)	性别 ♀ ♂	1d ♀ ♂	2d ♀ ♂	3d ♀ ♂	4d ♀ ♂	5d ♀ ♂	7d ♀ ♂	14d ♀ ♂	合计 ♀ ♂	中毒表现
15000	10 10	0 0	0 0	0 0	0 0	0 0	0 0	0 0	0 0	动物灌胃后无明显中毒表现，观察期内无动物死亡，处死动物大体解剖无明显异常.

（表头“1d”至“14d”列上方标注：动物死亡时间和数量）

注：染毒 96h 后出现迟发性新效应，应延长观察期至 3~4 周，记录另附。农药中毒表现详见下页。

LD_{50} 及 95%可信限（mg/kg b.wt.）：♀ >15000 ，♂ >15000

样品属性：☐农药 ☐食品 ☑保健食品 ☐化妆品 ☐消毒剂 ☐涉水产品 ☐化学品 ☐肥料 ☐其它：

毒性分级：无毒

检验人/记录人：吴俊　　审核人：[illegible]　　审核日期：2017 年 2 月 23 日
