# Supplementary material for: Safety evaluation of Aloe vera soft capsule in acute, subacute toxicity and genotoxicity study
Source: PLoS One. 2021 Mar 26;16(3):e0249356. doi: 10.1371/journal.pone.0249356 (PMC7997006; doi:10.1371/journal.pone.0249356)
Supplement: S2 File — (PDF) [file pone.0249356.s002.pdf]

食品（保健食品）Ames 试验原始记录

样品名称：芦荟软胶囊　　　　受理编号：G2020　20160028

生产日期或批号：2015 0904　　　　检验开始日期：2016 10.11

样品包装及形状：胶囊　　　　检验完成日期：2016 11-1

环境条件：房间号：813　温度：20℃～24℃　相对湿度：40～70%

一、菌株：经鉴定基因型符合要求的 TA97a、TA98、TA100 和 TA102（美国 MOLTOX），各菌株过夜培养细菌浓度在 10^9 或以上。

二、由β-萘黄酮和苯巴比妥联合诱导的大鼠肝匀浆，S_9 浓度为 10%。S_9 来源于齐氏生物科技有限公司。

三、受试液配制：

溶剂：水溶性：灭菌水□　脂溶性：二甲基亚砜☑、丙酮□、95%乙醇□、其它□

样品消毒：高压灭菌 121℃ 20min □ 106℃ 30min □　滤器除菌□　无菌直接取样☑

四、预试验：受试物剂量 5000 μg/皿，背景菌苔：正常☑ 减少□，TA [illegible] 产生杀/抑菌作。

五、正式试验：称取样品 50 mg，加入 DMSO 定容至 10 ml，作为最高剂量，再以该浓度溶液 5 倍稀释配制成次高剂量组受试液，准确吸取 1 ml 加入 [illegible] ml 定容至 5 ml，其余剂量依次方法梯度稀释。

六、剂量：样品：样品设 5 个剂量组，分别为 5000；1000；200；40；8 μg/皿

阳性对照组：不加 S_9 条件下，TA97a、TA98、TA102 为敌克松（50.0μg/皿）；TA100 为叠氮钠（1.5μg/皿）；加 S_9 条件下，TA97a、TA98、TA100 为 2-氨基芴（10.0μg/皿）；TA102 为 1,8-二羟基蒽醌（50.0μg/皿）。

七、试验步骤方法：(1)增菌培养：取营养肉汤培养基 5ml，加入无菌试管中，将主平板或冷冻保存的菌株培养物接种于营养肉汤培养基内，37℃振荡（100 次/min）培养 10 小时。（2）平板掺入法，实验时，将含 0.5mmol/L 组氨酸-0.5mmol/L 生物素溶液的顶层琼脂培养基 2.0ml 分装于试管中，45℃水浴中保温，然后分管依次加入试验菌株增菌液 0.1ml，受试物溶液 0.1ml 和 S_9 混合液 0.5ml（需代谢活化时），充分混匀，迅速倾入底层琼脂平板上，转动平板，使之分布均匀。水平放置待冷凝固化后，倒置于 37℃培养箱里孵育 48h。记数每皿回变菌落数。实验中，除设受试物各剂量组外，还同时设空白对照、溶剂对照、阳性诱变剂对照。每个剂量设平行样 3 个。重复试验二次。

八、菌落计数：计数前显微镜观察菌苔，用全自动菌落成像分析进行计数。

十、结果统计与评价：记录受试样品各剂量组、空白对照组自发回变、溶剂对照组及阳性对照组的每皿回变菌落数，并求平均值和标准差。受试样品的回变菌落数超过自发回变菌落数 2 倍以上，并呈剂量-效应关系判定检测结果为阳性。受试样品经四个试验菌株检测后，只要有一个试验菌株，无论在加 S_9 或不加 S_9 条件下为阳性者时，均可判定该受试样品 Ames 试验结果为阳性。四个试验菌株在加 S_9 和不加 S_9 条件下均为阴性，则可判定受试样品 Ames 试验结果为阴性。

十、主要仪器：生化培养箱(05-264)、全自动菌落成像系统(05-169)、压力蒸汽消毒器(05-259)、振荡水浴摇床(05-213)、电子天平编号 05-316

十一、结论：

□该受试物对标准测试菌 TA97a 呈现致突变性。
□该受试物对标准测试菌 TA98 呈现致突变性。
□该受试物对标准测试菌 TA100 呈现致突变性。
□该受试物对标准测试菌 TA102 呈现致突变性。
☑该受试物对标准测试菌 TA97a 未呈现致突变性。
□该受试物对标准测试菌 TA98 未呈现致突变性。
□该受试物对标准测试菌 TA100 未呈现致突变性。
□该受试物对标准测试菌 TA102 未呈现致突变性。

检验人/记录人：[illegible]　　审核人：[illegible]　　审核日期：2017年2月23日

样品编号：GZ02020160028AMES实验各菌株回变菌落计数原始数据对照（第一次）

组别	剂量 ug/皿	TA97a +S9	TA97a -S9	TA98 +S9	TA98 -S9	TA100 +S9	TA100 -S9	TA102 +S9	TA102 -S9
自发回变		123	102	35	29	125	124	265	251
		115	104	36	31	141	119	274	248
		124	98	34	32	139	134	268	256
	x	**121**	**101**	**35**	**31**	**135**	**126**	**269**	**252**
	s	**5**	**3**	**1**	**2**	**9**	**8**	**5**	**4**
溶剂对照 (DMSO)		141	129	41	30	135	126	268	256
		136	114	36	36	142	124	274	245
		134	108	32	35	139	138	281	284
	x	**137**	**117**	**36**	**34**	**139**	**129**	**274**	**262**
	s	**4**	**11**	**5**	**3**	**4**	**8**	**7**	**20**
样品组	8	141	119	40	36	135	132	269	256
		132	125	36	35	141	129	274	248
		120	134	38	41	136	136	257	265
	x	**131**	**126**	**38**	**37**	**137**	**132**	**267**	**256**
	s	**11**	**8**	**2**	**3**	**3**	**4**	**9**	**9**
	40	145	134	38	35	141	154	284	248
		132	145	37	34	135	141	278	297
		149	136	42	39	132	130	265	285
	x	**142**	**138**	**39**	**36**	**136**	**142**	**276**	**277**
	s	**9**	**6**	**3**	**3**	**5**	**12**	**10**	**26**
	200	141	126	42	32	125	115	263	264
		138	124	36	35	122	117	258	253
		148	151	39	36	129	138	271	249
	x	**142**	**134**	**39**	**34**	**125**	**123**	**264**	**255**
	s	**5**	**15**	**3**	**2**	**4**	**13**	**7**	**8**
	1000	162	118	40	36	165	132	268	252
		135	147	35	35	147	130	274	261
		124	132	38	37	142	121	281	264
	x	**140**	**132**	**38**	**36**	**151**	**128**	**274**	**259**
	s	**20**	**15**	**3**	**1**	**12**	**6**	**7**	**6**
	5000	169	124	41	32	142	142	284	246
		178	132	35	36	150	138	275	251
		177	110	32	29	139	132	265	260
	x	**175**	**122**	**36**	**32**	**144**	**137**	**275**	**252**
	s	**5**	**11**	**5**	**4**	**6**	**5**	**10**	**7**
阳性对照		2—AF	DEXON	2—AF	DEXON	2—AF	NAN3	1.8-DH/	DEXON
		1014	1125	868	1321	984	1214	1054	1136
		1245	1142	759	859	1014	1165	1187	1241
		745	1302	897	1045	1008	1135	985	859
	x	**1001**	**1190**	**841**	**1075**	**1002**	**1171**	**1075**	**1079**
	s	**250**	**98**	**73**	**232**	**16**	**40**	**103**	**197**

检验人/记录人 [signature]　　审核人：[signature]　　审核日期：2017.2.25

自发回变对照

SampleID					样品
20161021-901	123	0.804587	1	152.8734	TA97-s+
20161021-902	115	0.804587	1	142.9304	TA97-s+
20161021-903	124	0.804587	1	154.1162	TA97-s+
20161021-904	102	0.804587	1	126.7730	TA97-s-
20161021-905	104	0.804587	1	129.2588	TA97-s-
20161021-906	98	0.804587	1	121.8015	TA97-s-
20161021-907	35	0.804587	1	43.5006	TA98-s+
20161021-908	36	0.804587	1	44.7434	TA98-s+
20161021-909	34	0.804587	1	42.2577	TA98-s+
20161021-910	29	0.804587	1	36.0433	TA98-s-
20161021-911	31	0.804587	1	38.5291	TA98-s-
20161021-912	32	0.804587	1	39.7719	TA98-s-
20161021-913	125	0.804587	1	155.3591	TA100-s+
20161021-914	141	0.804587	1	175.2451	TA100-s+
20161021-915	139	0.804587	1	172.7593	TA100-s+
20161021-916	124	0.804587	1	154.1162	TA100-s-
20161021-917	119	0.804587	1	147.9019	TA100-s-
20161021-918	134	0.804587	1	166.5450	TA100-s-
20161021-919	265	0.804587	1	329.3613	TA102-s+
20161021-920	274	0.804587	1	340.5472	TA102-s+
20161021-921	268	0.804587	1	333.0900	TA102-s+
20161021-922	251	0.804587	1	311.9611	TA102-s-
20161021-923	248	0.804587	1	308.2325	TA102-s-
20161021-924	256	0.804587	1	318.1755	TA102-s-

阳性对照

SampleID					样品
20161021-951	1014	0.804587	1	1260.2732	Ta97-2-AF-s+
20161021-952	1245	0.804587	1	1547.3768	Ta97-2-AF-s+
20161021-953	745	0.804587	1	925.9404	Ta97-2-AF-s+
20161021-954	1125	0.804587	1	1398.2321	Ta97-DEXON-s-
20161021-955	1142	0.804587	1	1419.3609	Ta97-DEXON-s-
20161021-956	1302	0.804587	1	1618.2206	Ta97-DEXON-s-
20161021-957	868	0.804587	1	1078.8137	Ta98-2-AF-s+
20161021-958	759	0.804587	1	943.3406	Ta98-2-AF-s+
20161021-959	897	0.804587	1	1114.8570	Ta98-2-AF-s+
20161021-960	1321	0.804587	1	1641.8352	Ta98-DEXON-s-
20161021-961	859	0.804587	1	1067.6279	Ta98-DEXON-s-
20161021-962	1045	0.804587	1	1298.8022	Ta98-DEXON-s-
20161021-963	984	0.804587	1	1222.9870	Ta100-2-AF-s+
20161021-964	1014	0.804587	1	1260.2732	Ta100-2-AF-s+
20161021-965	1008	0.804587	1	1252.8159	Ta100-2-AF-s+
20161021-966	1214	0.804587	1	1508.8478	Ta100-NAN3-s-
20161021-967	1165	0.804587	1	1447.9470	Ta100-NAN3-s-
20161021-968	1135	0.804587	1	1410.6608	Ta100-NAN3-s-
20161021-969	1054	0.804587	1	1309.9881	Ta102-DHAQ-s+
20161021-970	1187	0.804587	1	1475.2902	Ta102-DHAQ-s+
20161021-971	985	0.804587	1	1224.2299	Ta102-DHAQ-s+
20161021-972	1136	0.804587	1	1411.9037	Ta102-DEXON-s-
20161021-973	1241	0.804587	1	1542.4053	Ta102-DEXON-s-
20161021-974	859	0.804587	1	1067.6279	Ta102-DEXON-s-

溶剂对照

20161021-925	141	0.804587	1	175.2451	TA97-DMSO-s+
20161021-926	136	0.804587	1	169.0307	TA97-DMSO-s+
20161021-927	134	0.804587	1	166.5450	TA97-DMSO-s+
20161021-928	129	0.804587	1	160.3306	TA97-DMSO-s-
20161021-929	114	0.804587	1	141.6875	TA97-DMSO-s-
20161021-930	108	0.804587	1	134.2303	TA97-DMSO-s-
20161021-931	41	0.804587	1	50.9578	TA98-DMSO-s+
20161021-932	36	0.804587	1	44.7434	TA98-DMSO-s+
20161021-933	32	0.804587	1	39.7719	TA98-DMSO-s+
20161021-934	30	0.804587	1	37.2862	TA98-DMSO-s-
20161021-935	36	0.804587	1	44.7434	TA98-DMSO-s-
20161021-936	35	0.804587	1	43.5006	TA98-DMSO-s-
20161021-937	135	0.804587	1	167.7878	TA100-DMSO-s+
20161021-938	142	0.804587	1	176.4880	TA100-DMSO-s+
20161021-939	139	0.804587	1	172.7593	TA100-DMSO-s+
20161021-940	126	0.804587	1	156.6020	TA100-DMSO-s-
20161021-941	124	0.804587	1	154.1162	TA100-DMSO-s-
20161021-942	138	0.804587	1	171.5165	TA100-DMSO-s-
20161021-943	268	0.804587	1	333.0900	TA102-DMSO-s+
20161021-944	274	0.804587	1	340.5472	TA102-DMSO-s+
20161021-945	281	0.804587	1	349.2473	TA102-DMSO-s+
20161021-946	256	0.804587	1	318.1755	TA102-DMSO-s-
20161021-947	245	0.804587	1	304.5039	TA102-DMSO-s-
20161021-948	284	0.804587	1	352.9759	TA102-DMSO-s-

	预实验		
	1#		2#
TA97-	113		105
TA97+	134		129
TA98-	41		39
TA98+	37		42
TA100-	142		170
TA100+	151		154
TA102-	271		253
TA102+	262		260
溶解度			可
有无污染			无
有无抑菌			无
确定浓度			5000

检验人 [signature]　　审核人 [signature]　　审核日期：2017年2.23

样品编号：GZ02020160028AMES实验各菌株回变菌落计数原始数据对照（第一次）2016-10-21 第8页 共89页

SampleID					样品	SampleID					样品
20161021-601	141	0.804587	1	175.2451	GZ02020160028-TA97-8-s+	20161021-661	135	0.804587	1	167.7878	GZ02020160028-TA100-8-s+
20161021-602	132	0.804587	1	164.0592	GZ02020160028-TA97-8-s+	20161021-662	141	0.804587	1	175.2451	GZ02020160028-TA100-8-s+
20161021-603	120	0.804587	1	149.1448	GZ02020160028-TA97-8-s+	20161021-663	136	0.804587	1	169.0307	GZ02020160028-TA100-8-s+
20161021-604	119	0.804587	1	147.9019	GZ02020160028-TA97-8-s-	20161021-664	132	0.804587	1	164.0592	GZ02020160028-TA100-8-s-
20161021-605	125	0.804587	1	155.3591	GZ02020160028-TA97-8-s-	20161021-665	129	0.804587	1	160.3306	GZ02020160028-TA100-8-s-
20161021-606	134	0.804587	1	166.5450	GZ02020160028-TA97-8-s-	20161021-666	136	0.804587	1	169.0307	GZ02020160028-TA100-8-s-
20161021-607	145	0.804587	1	180.2166	GZ02020160028-TA97-40-s+	20161021-667	141	0.804587	1	175.2451	GZ02020160028-TA100-40-s+
20161021-608	132	0.804587	1	164.0592	GZ02020160028-TA97-40-s+	20161021-668	135	0.804587	1	167.7878	GZ02020160028-TA100-40-s+
20161021-609	149	0.804587	1	185.1881	GZ02020160028-TA97-40-s+	20161021-669	132	0.804587	1	164.0592	GZ02020160028-TA100-40-s+
20161021-610	134	0.804587	1	166.5450	GZ02020160028-TA97-40-s-	20161021-670	154	0.804587	1	191.4024	GZ02020160028-TA100-40-s-
20161021-611	145	0.804587	1	180.2166	GZ02020160028-TA97-40-s-	20161021-671	141	0.804587	1	175.2451	GZ02020160028-TA100-40-s-
20161021-612	136	0.804587	1	169.0307	GZ02020160028-TA97-40-s-	20161021-672	130	0.804587	1	161.5735	GZ02020160028-TA100-40-s-
20161021-613	141	0.804587	1	175.2451	GZ02020160028-TA97-200-s+	20161021-673	125	0.804587	1	155.3591	GZ02020160028-TA100-200-s+
20161021-614	138	0.804587	1	171.5165	GZ02020160028-TA97-200-s+	20161021-674	122	0.804587	1	151.6305	GZ02020160028-TA100-200-s+
20161021-615	148	0.804587	1	183.9452	GZ02020160028-TA97-200-s+	20161021-675	129	0.804587	1	160.3306	GZ02020160028-TA100-200-s+
20161021-616	126	0.804587	1	156.6020	GZ02020160028-TA97-200-s-	20161021-676	115	0.804587	1	142.9304	GZ02020160028-TA100-200-s-
20161021-617	124	0.804587	1	154.1162	GZ02020160028-TA97-200-s-	20161021-677	117	0.804587	1	145.4161	GZ02020160028-TA100-200-s-
20161021-618	151	0.804587	1	187.6738	GZ02020160028-TA97-200-s-	20161021-678	138	0.804587	1	171.5165	GZ02020160028-TA100-200-s-
20161021-619	162	0.804587	1	201.3454	GZ02020160028-TA97-1000-s+	20161021-679	165	0.804587	1	205.0740	GZ02020160028-TA100-1000-s+
20161021-620	135	0.804587	1	167.7878	GZ02020160028-TA97-1000-s+	20161021-680	147	0.804587	1	182.7023	GZ02020160028-TA100-1000-s+
20161021-621	124	0.804587	1	154.1162	GZ02020160028-TA97-1000-s+	20161021-681	142	0.804587	1	176.4880	GZ02020160028-TA100-1000-s+
20161021-622	118	0.804587	1	146.6590	GZ02020160028-TA97-1000-s-	20161021-682	132	0.804587	1	164.0592	GZ02020160028-TA100-1000-s-
20161021-623	147	0.804587	1	182.7023	GZ02020160028-TA97-1000-s-	20161021-683	130	0.804587	1	161.5735	GZ02020160028-TA100-1000-s-
20161021-624	132	0.804587	1	164.0592	GZ02020160028-TA97-1000-s-	20161021-684	121	0.804587	1	150.3876	GZ02020160028-TA100-1000-s-
20161021-625	169	0.804587	1	210.0455	GZ02020160028-TA97-5000-s+	20161021-685	142	0.804587	1	176.4880	GZ02020160028-TA100-5000-s+
20161021-626	178	0.804587	1	221.2314	GZ02020160028-TA97-5000-s+	20161021-686	150	0.804587	1	186.4309	GZ02020160028-TA100-5000-s+
20161021-627	177	0.804587	1	219.9885	GZ02020160028-TA97-5000-s+	20161021-687	139	0.804587	1	172.7593	GZ02020160028-TA100-5000-s+
20161021-628	124	0.804587	1	154.1162	GZ02020160028-TA97-5000-s-	20161021-688	142	0.804587	1	176.4880	GZ02020160028-TA100-5000-s-
20161021-629	132	0.804587	1	164.0592	GZ02020160028-TA97-5000-s-	20161021-689	138	0.804587	1	171.5165	GZ02020160028-TA100-5000-s-
20161021-630	110	0.804587	1	136.7160	GZ02020160028-TA97-5000-s-	20161021-690	132	0.804587	1	164.0592	GZ02020160028-TA100-5000-s-
20161021-631	40	0.804587	1	49.7149	GZ02020160028-TA98-8-s+	20161021-691	269	0.804587	1	334.3328	GZ02020160028-TA102-8-s+
20161021-632	36	0.804587	1	44.7434	GZ02020160028-TA98-8-s+	20161021-692	274	0.804587	1	340.5472	GZ02020160028-TA102-8-s+
20161021-633	38	0.804587	1	47.2292	GZ02020160028-TA98-8-s+	20161021-693	257	0.804587	1	319.4184	GZ02020160028-TA102-8-s+
20161021-634	36	0.804587	1	44.7434	GZ02020160028-TA98-8-s-	20161021-694	256	0.804587	1	318.1755	GZ02020160028-TA102-8-s-
20161021-635	35	0.804587	1	43.5006	GZ02020160028-TA98-8-s-	20161021-695	248	0.804587	1	308.2325	GZ02020160028-TA102-8-s-
20161021-636	41	0.804587	1	50.9578	GZ02020160028-TA98-8-s-	20161021-696	265	0.804587	1	329.3613	GZ02020160028-TA102-8-s-
20161021-637	38	0.804587	1	47.2292	GZ02020160028-TA98-40-s+	20161021-697	284	0.804587	1	352.9759	GZ02020160028-TA102-40-s+
20161021-638	37	0.804587	1	45.9863	GZ02020160028-TA98-40-s+	20161021-698	278	0.804587	1	345.5187	GZ02020160028-TA102-40-s+
20161021-639	42	0.804587	1	52.2007	GZ02020160028-TA98-40-s+	20161021-699	265	0.804587	1	329.3613	GZ02020160028-TA102-40-s+
20161021-640	35	0.804587	1	43.5006	GZ02020160028-TA98-40-s-	20161021-700	248	0.804587	1	308.2325	GZ02020160028-TA102-40-s-
20161021-641	34	0.804587	1	42.2577	GZ02020160028-TA98-40-s-	20161021-701	297	0.804587	1	369.1333	GZ02020160028-TA102-40-s-
20161021-642	39	0.804587	1	48.4720	GZ02020160028-TA98-40-s-	20161021-702	285	0.804587	1	354.2188	GZ02020160028-TA102-40-s-
20161021-643	42	0.804587	1	52.2007	GZ02020160028-TA98-200-s+	20161021-703	263	0.804587	1	326.8756	GZ02020160028-TA102-200-s+
20161021-644	36	0.804587	1	44.7434	GZ02020160028-TA98-200-s+	20161021-704	258	0.804587	1	320.6612	GZ02020160028-TA102-200-s+
20161021-645	39	0.804587	1	48.4720	GZ02020160028-TA98-200-s+	20161021-705	271	0.804587	1	336.8186	GZ02020160028-TA102-200-s+
20161021-646	32	0.804587	1	39.7719	GZ02020160028-TA98-200-s-	20161021-706	264	0.804587	1	328.1185	GZ02020160028-TA102-200-s-
20161021-647	35	0.804587	1	43.5006	GZ02020160028-TA98-200-s-	20161021-707	253	0.804587	1	314.4469	GZ02020160028-TA102-200-s-
20161021-648	36	0.804587	1	44.7434	GZ02020160028-TA98-200-s-	20161021-708	249	0.804587	1	309.4754	GZ02020160028-TA102-200-s-
20161021-649	40	0.804587	1	49.7149	GZ02020160028-TA98-1000-s+	20161021-709	268	0.804587	1	333.0900	GZ02020160028-TA102-1000-s+
20161021-650	35	0.804587	1	43.5006	GZ02020160028-TA98-1000-s+	20161021-710	274	0.804587	1	340.5472	GZ02020160028-TA102-1000-s+
20161021-651	38	0.804587	1	47.2292	GZ02020160028-TA98-1000-s+	20161021-711	281	0.804587	1	349.2473	GZ02020160028-TA102-1000-s+
20161021-652	36	0.804587	1	44.7434	GZ02020160028-TA98-1000-s-	20161021-712	252	0.804587	1	313.2040	GZ02020160028-TA102-1000-s-
20161021-653	35	0.804587	1	43.5006	GZ02020160028-TA98-1000-s-	20161021-713	261	0.804587	1	324.3898	GZ02020160028-TA102-1000-s-
20161021-654	37	0.804587	1	45.9863	GZ02020160028-TA98-1000-s-	20161021-714	264	0.804587	1	328.1185	GZ02020160028-TA102-1000-s-
20161021-655	41	0.804587	1	50.9578	GZ02020160028-TA98-5000-s+	20161021-715	284	0.804587	1	352.9759	GZ02020160028-TA102-5000-s+
20161021-656	35	0.804587	1	43.5006	GZ02020160028-TA98-5000-s+	20161021-716	275	0.804587	1	341.7901	GZ02020160028-TA102-5000-s+
20161021-657	32	0.804587	1	39.7719	GZ02020160028-TA98-5000-s+	20161021-717	265	0.804587	1	329.3613	GZ02020160028-TA102-5000-s+
20161021-658	32	0.804587	1	39.7719	GZ02020160028-TA98-5000-s-	20161021-718	246	0.804587	1	305.7467	GZ02020160028-TA102-5000-s-
20161021-659	36	0.804587	1	44.7434	GZ02020160028-TA98-5000-s-	20161021-719	251	0.804587	1	311.9611	GZ02020160028-TA102-5000-s-
20161021-660	29	0.804587	1	36.0433	GZ02020160028-TA98-5000-s-	20161021-720	260	0.804587	1	323.1470	GZ02020160028-TA102-5000-s-

检验人 [illegible]　　审核人 [illegible]　　审核日期：2017年 2月 25日

样品编号：GZ02020160028AMES实验各菌株回变菌落计数原始数据对照（第二次）

组别	剂量 ug/皿	TA97a +S9	TA97a -S9	TA98 +S9	TA98 -S9	TA100 +S9	TA100 -S9	TA102 +S9	TA102 -S9
自发回变		132	124	32	30	152	114	274	254
		129	115	36	26	134	129	265	262
		114	114	29	29	128	130	281	269
	**x**	**125**	**118**	**32**	**28**	**138**	**124**	**273**	**262**
	**s**	**10**	**6**	**4**	**2**	**12**	**9**	**8**	**8**
溶剂对照		151	130	36	35	141	140	268	246
(DMSO)		142	125	34	32	135	119	274	261
		144	118	35	29	139	120	258	261
	**x**	**146**	**124**	**35**	**32**	**138**	**126**	**267**	**256**
	**s**	**5**	**6**	**1**	**3**	**3**	**12**	**8**	**9**
样品组	8	132	141	31	41	136	121	284	260
		136	132	36	32	132	134	265	257
		137	136	35	32	125	130	258	263
	**x**	**135**	**136**	**34**	**35**	**131**	**128**	**269**	**260**
	**s**	**3**	**5**	**3**	**5**	**6**	**7**	**13**	**3**
	40	149	135	36	34	145	132	258	264
		146	136	35	35	141	132	274	259
		144	141	39	32	130	125	250	231
	**x**	**146**	**137**	**37**	**34**	**139**	**130**	**261**	**251**
	**s**	**3**	**3**	**2**	**2**	**8**	**4**	**12**	**18**
	200	142	132	36	39	138	144	271	263
		136	136	38	35	145	132	258	254
		137	148	34	32	157	138	263	250
	**x**	**138**	**139**	**36**	**35**	**147**	**138**	**264**	**256**
	**s**	**3**	**8**	**2**	**4**	**10**	**6**	**7**	**7**
	1000	154	132	41	30	145	126	278	256
		148	131	35	26	147	127	264	284
		142	140	32	29	146	141	281	241
	**x**	**148**	**134**	**36**	**28**	**146**	**131**	**274**	**260**
	**s**	**6**	**5**	**5**	**2**	**1**	**8**	**9**	**22**
	5000	150	132	32	29	132	130	263	246
		142	131	38	36	131	136	253	244
		145	136	37	35	149	141	258	235
	**x**	**146**	**133**	**36**	**33**	**137**	**136**	**258**	**242**
	**s**	**4**	**3**	**3**	**4**	**10**	**6**	**5**	**6**
阳性对照		2—AF	DEXON	2—AF	DEXON	2—AF	NAN3	1.8-DHA	DEXON
		985	1241	956	1263	854	1325	1198	1063
		1045	1185	847	968	952	1041	1241	1258
		1147	1163	1014	1145	1147	1148	1004	956
	**x**	**1059**	**1196**	**939**	**1125**	**984**	**1171**	**1148**	**1092**
	**s**	**82**	**40**	**85**	**148**	**149**	**143**	**126**	**153**

检验人/记录人 [illegible]　　审核人：[illegible]　　审核日期：217.2.23

落计数原始数据对照（第二次）2016-10-28 第6页 共7页

SampleID					样品	SampleID					样品
自发回变对照						阳性对照					
20161028-901	132	0.804587	1	164.0592	TA97-s+	20161028-951	985	0.804587	1	1224.2299	Ta97-2-AF-s+
20161028-902	129	0.804587	1	160.3306	TA97-s+	20161028-952	1045	0.804587	1	1298.8022	Ta97-2-AF-s+
20161028-903	114	0.804587	1	141.6875	TA97-s-	20161028-953	1147	0.804587	1	1425.5753	Ta97-2-AF-s+
20161028-904	124	0.804587	1	154.1162	TA97-s-	20161028-954	1241	0.804587	1	1542.4053	Ta97-DEXON-s-
20161028-905	115	0.804587	1	142.9304	TA97-s-	20161028-955	1185	0.804587	1	1472.8045	Ta97-DEXON-s-
20161028-906	114	0.804587	1	141.6875	TA97-s-	20161028-956	1163	0.804587	1	1445.4612	Ta97-DEXON-s-
20161028-907	32	0.804587	1	39.7719	TA98-s+	20161028-957	956	0.804587	1	1188.1865	Ta98-2-AF-s+
20161028-908	36	0.804587	1	44.7434	TA98-s+	20161028-958	847	0.804587	1	1052.7134	Ta98-2-AF-s+
20161028-909	29	0.804587	1	36.0433	TA98-s+	20161028-959	1014	0.804587	1	1260.2732	Ta98-2-AF-s+
20161028-910	30	0.804587	1	37.2862	TA98-s-	20161028-960	1263	0.804587	1	1569.7485	Ta98-DEXON-s-
20161028-911	26	0.804587	1	32.3147	TA98-s-	20161028-961	968	0.804587	1	1203.1010	Ta98-DEXON-s-
20161028-912	29	0.804587	1	36.0433	TA98-s-	20161028-962	1145	0.804587	1	1423.0895	Ta98-DEXON-s-
20161028-913	152	0.804587	1	188.9167	TA100-s+	20161028-963	854	0.804587	1	1061.4135	Ta100-2-AF-s+
20161028-914	134	0.804587	1	166.5450	TA100-s+	20161028-964	952	0.804587	1	1183.2151	Ta100-2-AF-s+
20161028-915	128	0.804587	1	159.0877	TA100-s+	20161028-965	1147	0.804587	1	1425.5753	Ta100-2-AF-s+
20161028-916	114	0.804587	1	141.6875	TA100-s-	20161028-966	1325	0.804587	1	1646.8067	Ta100-NAN3-s-
20161028-917	129	0.804587	1	160.3306	TA100-s-	20161028-967	1041	0.804587	1	1293.8307	Ta100-NAN3-s-
20161028-918	130	0.804587	1	161.5735	TA100-s-	20161028-968	1148	0.804587	1	1426.8182	Ta100-NAN3-s-
20161028-919	274	0.804587	1	340.5472	TA102-s+	20161028-969	1198	0.804587	1	1488.9618	Ta102-DHAQ-s+
20161028-920	265	0.804587	1	329.3613	TA102-s+	20161028-970	1241	0.804587	1	1542.4053	Ta102-DHAQ-s+
20161028-921	281	0.804587	1	349.2473	TA102-s+	20161028-971	1004	0.804587	1	1247.8444	Ta102-DHAQ-s+
20161028-922	254	0.804587	1	315.6897	TA102-s-	20161028-972	1063	0.804587	1	1321.1740	Ta102-DEXON-s-
20161028-923	262	0.804587	1	325.6327	TA102-s-	20161028-973	1258	0.804587	1	1563.5342	Ta102-DEXON-s-
20161028-924	269	0.804587	1	334.3328	TA102-s-	20161028-974	956	0.804587	1	1188.1865	Ta102-DEXON-s-

溶剂对照

20161028-925	151	0.804587	1	187.6738	TA97-DMSO-s+
20161028-926	142	0.804587	1	176.4880	TA97-DMSO-s+
20161028-927	144	0.804587	1	178.9737	TA97-DMSO-s+
20161028-928	130	0.804587	1	161.5735	TA97-DMSO-s-
20161028-929	125	0.804587	1	155.3591	TA97-DMSO-s-
20161028-930	118	0.804587	1	146.6590	TA97-DMSO-s-
20161028-931	36	0.804587	1	44.7434	TA98-DMSO-s+
20161028-932	34	0.804587	1	42.2577	TA98-DMSO-s+
20161028-933	35	0.804587	1	43.5006	TA98-DMSO-s+
20161028-934	35	0.804587	1	43.5006	TA98-DMSO-s-
20161028-935	32	0.804587	1	39.7719	TA98-DMSO-s-
20161028-936	29	0.804587	1	36.0433	TA98-DMSO-s-
20161028-937	141	0.804587	1	175.2451	TA100-DMSO-s+
20161028-938	135	0.804587	1	167.7878	TA100-DMSO-s+
20161028-939	139	0.804587	1	172.7593	TA100-DMSO-s+
20161028-940	140	0.804587	1	174.0022	TA100-DMSO-s-
20161028-941	119	0.804587	1	147.9019	TA100-DMSO-s-
20161028-942	120	0.804587	1	149.1448	TA100-DMSO-s-
20161028-943	268	0.804587	1	333.0900	TA102-DMSO-s+
20161028-944	274	0.804587	1	340.5472	TA102-DMSO-s+
20161028-945	258	0.804587	1	320.6612	TA102-DMSO-s+
20161028-946	246	0.804587	1	305.7467	TA102-DMSO-s-
20161028-947	261	0.804587	1	324.3898	TA102-DMSO-s-
20161028-948	261	0.804587	1	324.3898	TA102-DMSO-s-

	预实验		
	1#		2#
TA97-			
TA97+			
TA98-			
TA98+			
TA100-			
TA100+			
TA102-			
TA102+			
溶解度			
有无污染			
有无抑菌			
确定浓度			

检验人 [signature]　　审核人 [signature]　　审核日期：2017年2.23

样品编号：GZ02020160028AMES实验各菌株回变菌落计数原始数据对照（第二次）2016-10-28 第11页 共[illegible]9页

SampleID					样品	SampleID					样品
20161028-601	132	0.804587	1	164.0592	GZ02020160028-TA97-8-s+	20161028-661	136	0.804587	1	169.0307	GZ02020160028-TA100-8-s+
20161028-602	136	0.804587	1	169.0307	GZ02020160028-TA97-8-s+	20161028-662	132	0.804587	1	164.0592	GZ02020160028-TA100-8-s+
20161028-603	137	0.804587	1	170.2736	GZ02020160028-TA97-8-s+	20161028-663	125	0.804587	1	155.3591	GZ02020160028-TA100-8-s+
20161028-604	141	0.804587	1	175.2451	GZ02020160028-TA97-8-s-	20161028-664	121	0.804587	1	150.3876	GZ02020160028-TA100-8-s-
20161028-605	132	0.804587	1	164.0592	GZ02020160028-TA97-8-s-	20161028-665	134	0.804587	1	166.5450	GZ02020160028-TA100-8-s-
20161028-606	136	0.804587	1	169.0307	GZ02020160028-TA97-8-s-	20161028-666	130	0.804587	1	161.5735	GZ02020160028-TA100-8-s-
20161028-607	149	0.804587	1	185.1881	GZ02020160028-TA97-40-s+	20161028-667	145	0.804587	1	180.2166	GZ02020160028-TA100-40-s+
20161028-608	146	0.804587	1	181.4595	GZ02020160028-TA97-40-s+	20161028-668	141	0.804587	1	175.2451	GZ02020160028-TA100-40-s+
20161028-609	144	0.804587	1	178.9737	GZ02020160028-TA97-40-s+	20161028-669	130	0.804587	1	161.5735	GZ02020160028-TA100-40-s+
20161028-610	135	0.804587	1	167.7878	GZ02020160028-TA97-40-s-	20161028-670	132	0.804587	1	164.0592	GZ02020160028-TA100-40-s-
20161028-611	136	0.804587	1	169.0307	GZ02020160028-TA97-40-s-	20161028-671	132	0.804587	1	164.0592	GZ02020160028-TA100-40-s-
20161028-612	141	0.804587	1	175.2451	GZ02020160028-TA97-40-s-	20161028-672	125	0.804587	1	155.3591	GZ02020160028-TA100-40-s-
20161028-613	142	0.804587	1	176.4880	GZ02020160028-TA97-200-s+	20161028-673	138	0.804587	1	171.5165	GZ02020160028-TA100-200-s+
20161028-614	136	0.804587	1	169.0307	GZ02020160028-TA97-200-s+	20161028-674	145	0.804587	1	180.2166	GZ02020160028-TA100-200-s+
20161028-615	137	0.804587	1	170.2736	GZ02020160028-TA97-200-s+	20161028-675	157	0.804587	1	195.1311	GZ02020160028-TA100-200-s+
20161028-616	132	0.804587	1	164.0592	GZ02020160028-TA97-200-s-	20161028-676	144	0.804587	1	178.9737	GZ02020160028-TA100-200-s-
20161028-617	136	0.804587	1	169.0307	GZ02020160028-TA97-200-s-	20161028-677	132	0.804587	1	164.0592	GZ02020160028-TA100-200-s-
20161028-618	148	0.804587	1	183.9452	GZ02020160028-TA97-200-s-	20161028-678	138	0.804587	1	171.5165	GZ02020160028-TA100-200-s-
20161028-619	154	0.804587	1	191.4024	GZ02020160028-TA97-1000-s+	20161028-679	145	0.804587	1	180.2166	GZ02020160028-TA100-1000-s+
20161028-620	148	0.804587	1	183.9452	GZ02020160028-TA97-1000-s+	20161028-680	147	0.804587	1	182.7023	GZ02020160028-TA100-1000-s+
20161028-621	142	0.804587	1	176.4880	GZ02020160028-TA97-1000-s+	20161028-681	146	0.804587	1	181.4595	GZ02020160028-TA100-1000-s+
20161028-622	132	0.804587	1	164.0592	GZ02020160028-TA97-1000-s-	20161028-682	126	0.804587	1	156.6020	GZ02020160028-TA100-1000-s-
20161028-623	131	0.804587	1	162.8164	GZ02020160028-TA97-1000-s-	20161028-683	127	0.804587	1	157.8449	GZ02020160028-TA100-1000-s-
20161028-624	140	0.804587	1	174.0022	GZ02020160028-TA97-1000-s-	20161028-684	141	0.804587	1	175.2451	GZ02020160028-TA100-1000-s-
20161028-625	150	0.804587	1	186.4309	GZ02020160028-TA97-5000-s+	20161028-685	132	0.804587	1	164.0592	GZ02020160028-TA100-5000-s+
20161028-626	142	0.804587	1	176.4880	GZ02020160028-TA97-5000-s+	20161028-686	131	0.804587	1	162.8164	GZ02020160028-TA100-5000-s+
20161028-627	145	0.804587	1	180.2166	GZ02020160028-TA97-5000-s+	20161028-687	149	0.804587	1	185.1881	GZ02020160028-TA100-5000-s+
20161028-628	132	0.804587	1	164.0592	GZ02020160028-TA97-5000-s-	20161028-688	130	0.804587	1	161.5735	GZ02020160028-TA100-5000-s-
20161028-629	131	0.804587	1	162.8164	GZ02020160028-TA97-5000-s-	20161028-689	136	0.804587	1	169.0307	GZ02020160028-TA100-5000-s-
20161028-630	136	0.804587	1	169.0307	GZ02020160028-TA97-5000-s-	20161028-690	141	0.804587	1	175.2451	GZ02020160028-TA100-5000-s-
20161028-631	31	0.804587	1	38.5291	GZ02020160028-TA98-8-s+	20161028-691	284	0.804587	1	352.9759	GZ02020160028-TA102-8-s+
20161028-632	36	0.804587	1	44.7434	GZ02020160028-TA98-8-s+	20161028-692	265	0.804587	1	329.3613	GZ02020160028-TA102-8-s+
20161028-633	35	0.804587	1	43.5006	GZ02020160028-TA98-8-s+	20161028-693	258	0.804587	1	320.6612	GZ02020160028-TA102-8-s+
20161028-634	41	0.804587	1	50.9578	GZ02020160028-TA98-8-s-	20161028-694	260	0.804587	1	323.1470	GZ02020160028-TA102-8-s-
20161028-635	32	0.804587	1	39.7719	GZ02020160028-TA98-8-s-	20161028-695	257	0.804587	1	319.4184	GZ02020160028-TA102-8-s-
20161028-636	32	0.804587	1	39.7719	GZ02020160028-TA98-8-s-	20161028-696	263	0.804587	1	326.8756	GZ02020160028-TA102-8-s-
20161028-637	36	0.804587	1	44.7434	GZ02020160028-TA98-40-s+	20161028-697	258	0.804587	1	320.6612	GZ02020160028-TA102-40-s+
20161028-638	35	0.804587	1	43.5006	GZ02020160028-TA98-40-s+	20161028-698	274	0.804587	1	340.5472	GZ02020160028-TA102-40-s+
20161028-639	39	0.804587	1	48.4720	GZ02020160028-TA98-40-s+	20161028-699	250	0.804587	1	310.7182	GZ02020160028-TA102-40-s+
20161028-640	34	0.804587	1	42.2577	GZ02020160028-TA98-40-s-	20161028-700	264	0.804587	1	328.1185	GZ02020160028-TA102-40-s-
20161028-641	35	0.804587	1	43.5006	GZ02020160028-TA98-40-s-	20161028-701	259	0.804587	1	321.9041	GZ02020160028-TA102-40-s-
20161028-642	32	0.804587	1	39.7719	GZ02020160028-TA98-40-s-	20161028-702	231	0.804587	1	287.1037	GZ02020160028-TA102-40-s-
20161028-643	36	0.804587	1	44.7434	GZ02020160028-TA98-200-s+	20161028-703	271	0.804587	1	336.8186	GZ02020160028-TA102-200-s+
20161028-644	38	0.804587	1	47.2292	GZ02020160028-TA98-200-s+	20161028-704	258	0.804587	1	320.6612	GZ02020160028-TA102-200-s+
20161028-645	34	0.804587	1	42.2577	GZ02020160028-TA98-200-s+	20161028-705	263	0.804587	1	326.8756	GZ02020160028-TA102-200-s+
20161028-646	39	0.804587	1	48.4720	GZ02020160028-TA98-200-s-	20161028-706	263	0.804587	1	326.8756	GZ02020160028-TA102-200-s-
20161028-647	35	0.804587	1	43.5006	GZ02020160028-TA98-200-s-	20161028-707	254	0.804587	1	315.6897	GZ02020160028-TA102-200-s-
20161028-648	32	0.804587	1	39.7719	GZ02020160028-TA98-200-s-	20161028-708	250	0.804587	1	310.7182	GZ02020160028-TA102-200-s-
20161028-649	41	0.804587	1	50.9578	GZ02020160028-TA98-1000-s+	20161028-709	278	0.804587	1	345.5187	GZ02020160028-TA102-1000-s+
20161028-650	35	0.804587	1	43.5006	GZ02020160028-TA98-1000-s+	20161028-710	264	0.804587	1	328.1185	GZ02020160028-TA102-1000-s+
20161028-651	32	0.804587	1	39.7719	GZ02020160028-TA98-1000-s+	20161028-711	281	0.804587	1	349.2473	GZ02020160028-TA102-1000-s+
20161028-652	30	0.804587	1	37.2862	GZ02020160028-TA98-1000-s-	20161028-712	256	0.804587	1	318.1755	GZ02020160028-TA102-1000-s-
20161028-653	26	0.804587	1	32.3147	GZ02020160028-TA98-1000-s-	20161028-713	284	0.804587	1	352.9759	GZ02020160028-TA102-1000-s-
20161028-654	29	0.804587	1	36.0433	GZ02020160028-TA98-1000-s-	20161028-714	241	0.804587	1	299.5324	GZ02020160028-TA102-1000-s-
20161028-655	32	0.804587	1	39.7719	GZ02020160028-TA98-5000-s+	20161028-715	263	0.804587	1	326.8756	GZ02020160028-TA102-5000-s+
20161028-656	38	0.804587	1	47.2292	GZ02020160028-TA98-5000-s+	20161028-716	253	0.804587	1	314.4469	GZ02020160028-TA102-5000-s+
20161028-657	37	0.804587	1	45.9863	GZ02020160028-TA98-5000-s+	20161028-717	258	0.804587	1	320.6612	GZ02020160028-TA102-5000-s+
20161028-658	29	0.804587	1	36.0433	GZ02020160028-TA98-5000-s-	20161028-718	246	0.804587	1	305.7467	GZ02020160028-TA102-5000-s-
20161028-659	36	0.804587	1	44.7434	GZ02020160028-TA98-5000-s-	20161028-719	244	0.804587	1	303.2610	GZ02020160028-TA102-5000-s-
20161028-660	35	0.804587	1	43.5006	GZ02020160028-TA98-5000-s-	20161028-720	235	0.804587	1	292.0751	GZ02020160028-TA102-5000-s-

检验人 [illegible]　　审核人 [illegible]　　审核日期：2017年 2月23日
