# Supplementary material for: Safety evaluation of Aloe vera soft capsule in acute, subacute toxicity and genotoxicity study
Source: PLoS One. 2021 Mar 26;16(3):e0249356. doi: 10.1371/journal.pone.0249356 (PMC7997006; doi:10.1371/journal.pone.0249356)
Supplement: S3 File — (PDF) [file pone.0249356.s003.pdf]

小鼠骨髓微核试验原始记录

样品编号：( ) GZ02020160028　　检验日期：2016年10月17日—2016年10月31日

动物数量：50只，动物试验环境：1009室，温度22—24℃，相对湿度40—70%

主要仪器：电子秤（05-718），电子天平（05-268）

一、动物体重及分组：

组别	10000 mg/kg (1/__LD$_{50}$)					5000 mg/kg (1/__LD$_{50}$)					2500 mg/kg (1/__LD$_{50}$)				
编号	1	2	3	4	5	1	2	3	4	5	1	2	3	4	5
体重(g) ♀	26.1	25.7	26.6	27.3	25.6	26.4	27.0	26.1	25.9	27.7	26.2	27.4	25.9	26.2	25.3
♂	28.2	27.4	26.2	26.7	27.1	26.2	28.4	26.3	27.2	27.9	28.8	28.1	26.4	26.2	26.0

组别：溶剂对照（15 ml/kg）　阳性对照（环磷酰胺）40 mg/kg　环磷酰胺批号：WXBC0418V

组别	溶剂对照					阳性对照				
编号	1	2	3	4	5	1	2	3	4	5
体重(g) ♀	25.9	26.9	27.0	26.1	26.5	26.1	26.8	27.2	26.6	26.0
♂	27.7	25.9	26.3	26.6	28.3	28.9	25.6	26.7	27.4	27.0

二、方法：本样品 LD$_{50}$ 值（mg/kg b.wt.）：♀ ＞15000　♂ ＞15000

	剂量（mg/kg）	样品(mg)	溶剂加至(ml)	灌胃量（ml/kg）	
样品	H 10000	10000	15	15（油为溶剂）	（√）
	M 5000	5000	15	20（水或胶为溶剂）	（ ）
	L 2500	2500	15		
阳性对照	环磷酰胺 40	20/40	10/20	20	（√）
溶剂对照	纯水（ ） 植物油（√） [illegible]	%医用淀粉（ ）	0.5%羧甲基纤维素钠（ ）	吐温 80（ ）	其它：

30h2 次给药，第 2 次给药后 6h 取股骨髓，涂片，Giemsa 染色，镜检和计数，每只动物 1000 个嗜多染红细胞(PCE)，计数含微核的嗜多染红细胞，计算微核细胞率（‰），每只动物计数 200 个 PCE，记数同时所见的成熟红细胞（NCE），并计算 PCE/NCE 比值。

三、结果：见下表

组别	剂量 (mg/kg)	鼠号	PCE/NCE 1	2	3	4	5	PCE/NCE 值 ($\bar{x} \pm s$)	含微核的 PCE 数 1	2	3	4	5	微核细胞率 (‰，$\bar{x} \pm s$)
溶剂对照		♀	/	/	/	/	/	1.01±0.07	2	2	1	1	2	1.60±0.55
		♂	/	/	/	/	/	1.02±0.07	2	3	0	2	1	1.60±1.14
样品	H	♀	/	/	/	/	/	1.01±0.05	2	2	1	1	1	1.40±0.55
	H	♂	/	详见后页	/	/	/	1.03±0.07	2	2	1	1	1	1.40±0.55
	M	♀	/	/	/	/	/	0.98±0.04	3	1	1	1	2	1.60±0.89
	M	♂	/	/	/	/	/	1.03±0.03	1	1	3	0	2	1.40±1.14
	L	♀	/	/	/	/	/	0.97±0.03	2	1	2	1	1	1.40±0.55
	L	♂	/	/	/	/	/	1.00±0.04	2	1	3	1	1	1.60±0.89
阳性对照	40	♀	/	/	/	/	/	0.93±0.05	29	25	22	24	20	24.00±3.39
		♂	/	/	/	/	/	0.98±0.10	21	17	19	18	25	20.00±3.16

检验人/记录人：吴俊　　审核人：杨明　审核日期：2017年2月23日

	GZ02020160028微核阅片记录统计									
	雌性									
	含微核的PCE数									
	C	L	M	H	CP					
1	2	2	3	2	29					
2	2	1	1	2	25					
3	1	2	1	1	22					
4	1	1	1	1	24					
5	2	1	2	1	20					
X	1.60	1.40	1.60	1.40	24.00					
S	0.55	0.55	0.89	0.55	3.39					
SUM	8	7	8	7	120					
P		0.58	1	0.5796	9E-05					
	观察的PCE及NCE数									
	C		L		M		H		CP	
	PCE	NCE	PCE	NCE	PCE	NCE	PCE	NCE	PCE	NCE
1	214	200	205	215	209	215	206	197	201	232
2	200	192	215	223	214	227	224	215	221	242
3	206	199	210	207	206	198	233	239	200	217
4	206	230	210	215	214	210	215	230	210	209
5	204	200	216	230	211	223	207	200	224	235
SUM	1030	1021	1056	1090	1054	1073	1085	1081	1056	1135
	PCE/NCE值									
	C	L	M	H	CP					
1	1.07	0.95	0.97	1.05	0.87					
2	1.04	0.96	0.94	1.04	0.91					
3	1.04	1.01	1.04	0.97	0.92					
4	0.90	0.98	1.02	0.93	1.00					
5	1.02	0.94	0.95	1.04	0.95					
X	1.01	0.97	0.98	1.01	0.93					
S	0.07	0.03	0.04	0.05	0.05					

	GZ02020160028微核阅片记录统计									
	雄性									
	含微核的PCE数									
	C	L	M	H	CP					
	2	2	1	2	21					
	3	1	1	2	17					
	0	3	3	1	19					
	2	1	0	1	18					
	1	1	2	1	25					
X	1.60	1.60	1.40	1.40	20.00					
S	1.14	0.89	1.14	0.55	3.16					
SUM	8	8	7	7	100					
P		1	0.789	0.736	6E-05					
	观察的PCE及NCE数									
	C		L		M		H		CP	
	PCE	NCE	PCE	NCE	PCE	NCE	PCE	NCE	PCE	NCE
	220	197	224	231	230	220	233	216	200	216
	214	206	227	230	215	211	209	215	212	195
	232	240	206	201	200	204	225	207	215	244
	212	205	215	204	216	207	222	205	206	190
	200	215	217	223	217	204	211	223	200	221
SUM	1078	1063	1089	1089	1078	1046	1100	1066	1033	1066
	PCE/NCE值									
	C	L	M	H	CP					
1	1.12	0.97	1.05	1.08	0.93					
2	1.04	0.99	1.02	0.97	1.09					
3	0.97	1.02	0.98	1.09	0.88					
4	1.03	1.05	1.04	1.08	1.08					
5	0.93	0.97	1.06	0.95	0.90					
X	1.02	1.00	1.03	1.03	0.98					
S	0.07	0.04	0.03	0.07	0.10					

统计：　　　　审核：　　　　审核日期

05-037

样品编号：毒 6#28　　　读片日期：2006年10月28日　时-　时

微核试验读片结果记录

片号	染毒剂量(mg/kg)	动物性别	含微核的 PCE 数/1000 个 PCE(个)	含多微核的 PCE 数(个)	PCE/NCE
1	H	♀	T		206/197
2			T		224/215
3			一		233/239
4			一		215/230
5			一		207/200
1	M	♀	下		209/215
2			一		214/227
3			一		206/198
4			一		214/210
5			T		211/223
1	L	♀	T		205/215
2			一		215/223
3			T		210/207
4			一		210/215
5			一		216/230

读片人/记录人：[illegible]　　审核人：[illegible]　审核日期：2007年2月23日

样品编号：毒 GZ 28　　　　读片日期：2016年10月31日　时-　时

微核试验读片结果记录

片号	染毒剂量(mg/kg)	动物性别	含微核的 PCE 数/1000 个 PCE(个)	含多微核的 PCE 数(个)	PCE/NCE
1	H	♂	T		233/216
2			T		209/215
3			一		225/207
4			一		222/205
5			一		211/223
1	M	♂	一		230/220
2			一		215/211
3			下		200/204
4			0		216/207
5			T		217/204
1	L	♂	T		224/231
2			一		227/230
3			下		206/201
4			一		215/204
5			一		217/223

读片人/记录人：吴俊　　　　审核人：[illegible]　审核日期：2017年2月23日

样品编号：毒　阳性　　　　读片日期：2016年10月24 25日　　时-　　时

微核试验读片结果记录

片号	染毒剂量 (mg/kg)	动物性别	含微核的 PCE 数/1000 个 PCE(个)	含多微核的 PCE 数(个)	PCE/NCE
1	40mg/kg 环磷酰胺	♀	正正正正正下		201/232
2	40	♀	正正正正正		221/242
3	40	♀	正正正正丅		200/217
4	40	♀	正正正正下		210/209
5	40	♀	正正正正		224/235
1	40mg/kg 环磷酰胺	♂	正正正正一		200/216
2	40	♂	正正正丅		212/195
3	40	♂	正正正正		215/244
4	40	♂	正正正下		206/190
5	40	♂	正正正正正		200/221

读片人/记录人：吴[illegible]　　审核人：杨[illegible]　审核日期：2017年　2月　23日

样品编号：毒 油阴性　　　　读片日期：年10月26日-27 时- 时

微核试验读片结果记录

05-219

片号	染毒剂量 (mg/kg)	动物性别	含微核的 PCE 数/1000 个 PCE(个)	含多微核的 PCE 数(个)	PCE/NCE
1	色拉油 0	♀	T		214/200
2	0	♀	T		200/192
3	0	♀	一		206/199
4	0	♀	一		206/230
5	0	♀	T		204/200
1	0	♂	T		220/197
2	0	♂	F		214/206
3	0	♂	0		232/240
4	0	♂	T		212/205
5	0	♂	一		200/215

读片人/记录人：吴俊　　审核人：[illegible]　审核日期：[illegible]年 2月 23日
