# Supplementary material for: Safety evaluation of Aloe vera soft capsule in acute, subacute toxicity and genotoxicity study
Source: PLoS One. 2021 Mar 26;16(3):e0249356. doi: 10.1371/journal.pone.0249356 (PMC7997006; doi:10.1371/journal.pone.0249356)
Supplement: S4 File — (PDF) [file pone.0249356.s004.pdf]

小鼠睾丸染色体畸变试验原始记录

样品编号：GZ02020160028　　检验日期：2016年10月17日—2016年12月15日

一、动物数量：雄性25只，试验环境：1009室，温度20—24℃，相对湿度40—70%

编号	1	2	3	4	5	6	7	8	9	10	12	13	14	15	16	17	18	19	23	24	25	26	27	28	29
体重	26.2	27.1	25.4	25.6	27.0	26.1	26.9	27.7	26.2	25.9	28.1	27.4	26.2	25.4	27.0	26.4	27.3	28.2	25.7	26.0	25.9	27.7	27.5	28.0	26.7
组别	阴	阴	阴	阴	阴	低	低	低	低	低	中	中	中	中	中	高	高	高	高	高	阳	阳	阳	阳	阳

二、方法：本样品 LD_{50} 值（mg/kg b.wt.）：♀ >15000　♂ >15000

	剂量（mg/kg）	样品(mg)	溶剂加至(ml)	灌胃量（ml/kg）
样品	L 2500	2500	15	15 ~~10~~（油为溶剂）（✓）
	M 5000	5000	15	20（水或胶为溶剂）（ ）
	H 10000	10000	15	

阳性对照　丝裂霉素C 4mg加生理盐水至20mL，充分振匀，一次腹腔注射15h，10mL/kg·b.wt

溶剂对照　双蒸水（ ）植物油 色拉油（✓）___%医用淀粉（ ）0.5%羧甲基纤维素钠（ ）吐温80（ ）其它：

称取秋水仙素8mg加生理盐水至20mL混合均匀。各组动物于处死前6h腹腔注射秋水仙素4mg/kg，注射量为10mL/kg。

三、结果：

剂量 (mg/kg)	组别及片号	观察细胞数	性分	常分	断裂	断片	链状四价体	环状四价体	三价体	多价体	畸变细胞率（%）（$\bar{x}\pm s$）
阴性对照	0-1	100	[illegible]	T	0	0	0	0	0	0	0
	0-2	100	T	T	0	一	0	0	0	0	1
	0-3	100	T	T	0	0	0	0	0	0	0
	0-4	100	止	止	0	0	0	0	0	0	0
	0-5	100	下	T	0	一	0	0	0	0	1
	$\bar{x}\pm s$		3.0±0.9	2.4±0.8	0	0.4±0.5					0.4±0.5
2500 L	1-1	100	下	一	0	0	0	0	0	0	0
	1-2	100	止	T	0	一	0	0	0	0	1
	1-3	100	T	止	0	0	0	0	0	0	0
	1-4	100	下	T	0	一	0	0	0	0	1
	1-5	100	下	一	0	0	0	0	0	0	0
	$\bar{x}\pm s$		3.0±0.6	2.0±1.1	0	0.4±0.5					0.4±0.5
5000 M	2-1	100	止	一	0	0	0	0	0	0	0
	2-2	100	下	下	0	0	0	0	0	0	0
	2-3	100	止	一	0	0	0	0	0	0	0
	2-4	100	下	T	0	0	0	0	0	0	0
	2-5	100	T	止	0	一	0	0	0	0	1
	$\bar{x}\pm s$		3.2±0.7	2.2±1.2	0	0.2±0.4					0.2±0.4
10000 H	3-1	100	止	T	0	0	0	0	0	0	0
	3-2	100	止	止	0	一	0	0	0	0	1
	3-3	100	正	下	0	0	0	0	0	0	0
	3-4	100	T	下	0	0	0	0	0	0	0
	3-5	100	T	T	0	0	0	0	0	0	0
	$\bar{x}\pm s$		3.4±1.2	2.8±0.7	0	0.2±0.4					0.2±0.4
阳性对照 丝裂霉素C 2mg/kg	阳-1	100	正正	正下	0	正止	一	0	0	0	10
	阳-2	100	正T	正一	0	正正	0	0	0	0	10
	阳-3	100	正下	正	0	正下	0	0	0	0	8
	阳-4	100	正正	正一	0	正一	0	0	0	0	6
	阳-5	100	正止	正T	0	正止	0	0	0	0	9
	$\bar{x}\pm s$		8.8±1.3	6.4±1.0	0	8.4±1.4	0.2±0.4				8.6±1.5

检验人/记录人：吴俊　　审核人：[illegible]　　审核日期：2017年2月23日

样品编号：G20202016 0028　　　　读片日期：2016 年 11 月 22-23 日　时-　时

染色体畸变试验阅片结果记录

片号	染毒剂量 mg/kg	观察细胞数	性分（个）	常分（个）	畸变类型 断裂隙	断片	易位	微小体	[illegible]	畸变细胞数（个）
1	2500	100	下	一	0	0	0	0	0	0
2	2500	100	正	丅	0	一	0	0	0	1
3	2500	100	丅	正	0	0	0	0	0	0
4	2500	100	下	丅	0	一	0	0	0	1
5	2500	100	下	一	0	0	0	0	0	0
1	5000	100	正	一	0	0	0	0	0	0
2	5000	100	下	下	0	0	0	0	0	0
3	5000	100	正	一	0	0	0	0	0	0
4	5000	100	下	丅	0	0	0	0	0	0
5	5000	100	丅	正	0	一	0	0	0	1
1	10000	100	正	丅	0	0	0	0	0	0
2	10000	100	正	正	0	一	0	0	0	1
3	10000	100	正	下	0	0	0	0	0	0
4	10000	100	丅	下	0	0	0	0	0	0
5	10000	100	丅	丅	0	0	0	0	0	0

其它：包括缺失、内复制、插入、倒位、粉碎、环、易位、姐妹染色单体交换、三着丝粒等畸变类型

读片人/记录人：吴俊　　　　审核人：[illegible]　　　　审核日期：2017 年 2 月 23 日

样品编号：对照　　　　读片日期：2016年 12月 15日 9 时-　　时

染色体畸变试验阅片结果记录

片号	染毒剂量 mg/kg	观察细胞数	性分（个）	常分（个）	畸变类型					畸变细胞数（个）
					裂隙	断片	易位	微小体	链状畸[illegible]	
1	2MMC	100	正正	正下	0	正止	0	0	一	10
2	2MMC	100	正T	正一	0	正正	0	0	0	10
3	2MMC	100	正下	正	0	正下	0	0	0	8
4	2MMC	100	正正	正一	0	正一	0	0	0	6
5	2MMC	100	正止	正T	0	正止	0	0	0	9
1	0	100	止	T	0	0	0	0	0	0
2	0	100	T	T	0	一	0	0	0	1
3	0	100	T	T	0	0	0	0	0	0
4	0	100	止	止	0	0	0	0	0	0
5	0	100	下	T	0	一	0	0	0	1

其它：包括缺失、内复制、插入、倒位、粉碎、环、易位、姐妹染色单体交换、三着丝粒等畸变类型

读片人/记录人：[illegible]　　审核人：[illegible]　　审核日期：2017年 2月 23日
