# Supplementary material for: Safety evaluation of Aloe vera soft capsule in acute, subacute toxicity and genotoxicity study
Source: PLoS One. 2021 Mar 26;16(3):e0249356. doi: 10.1371/journal.pone.0249356 (PMC7997006; doi:10.1371/journal.pone.0249356)
Supplement: S5 File — (PDF) [file pone.0249356.s005.pdf]

(30)天喂养试验大鼠体重原始记录（g）

样品编号：G802020160028	喂养房间号：924
动物性别：♀	环境温度：20—24 ℃
动物体重秤编号：05-715	相对湿度：60—70 %

对照组							低剂量组						
周	0w	1w	2w	3w	4w	空腹	周	0w	1w	2w	3w	4w	空腹
日期	10.12	10.19	10.26	11.2	11.10	11.11	日期	10.12	10.19	10.26	11.2	11.10	11.11
1	77	128	166	189	220	207	1	80	127	161	178	205	192
2	89	134	182	195	230	221	2	78	132	171	189	221	208
3	85	142	188	226	257	248	3	80	128	162	195	211	204
4	85	133	181	213	243	236	4	84	130	168	192	214	210
5	82	123	165	185	209	206	5	78	126	171	191	218	204
6	84	111	180	200	227	220	6	83	129	172	190	221	212
7	85	128	164	180	203	198	7	83	126	159	185	218	207
8	80	134	176	194	230	221	8	86	136	175	208	236	233
9	76	125	159	186	210	203	9	80	121	160	177	209	199
10	88	128	160	180	201	196	10	87	145	191	218	262	243
11							11						
12							12						

中剂量组							高剂量组						
1	78	119	154	174	203	201	1	79	118	145	165	194	184
2	82	127	162	183	198	191	2	80	123	170	199	232	223
3	84	131	170	194	240	233	3	76	106	151	167	190	183
4	84	123	171	186	210	203	4	86	131	162	187	211	206
5	79	126	163	182	216	213	5	77	108	140	166	189	181
6	85	130	164	180	203	197	6	81	120	156	177	204	~~206~~ 193 吴俊
7	92	137	179	219	249	241	7	77	119	159	183	205	192
8	78	114	153	175	192	190	8	82	129	180	197	223	214
9	81	135	174	187	221	216	9	82	120	158	178	198	185
10	80	109	148	169	188	179	10	88	136	187	213	245	235
11							11						
12							12						

检验人/记录人：吴俊　　　　审核人：[illegible]　　　　日期：2017.2.23

(30)天喂养试验大鼠体重原始记录（g）

样品编号：G202020160028	喂养房间号：924
动物性别：♂	环境温度：20 — 24 ℃
动物体重秤编号：05-715	相对湿度：40—70 %

对照组

周	0w	1w	2w	3w	4w	空腹
日期	10.12	10.19	10.26	11.2	11.10	11.11
1	80	134	189	241	294	288
2	86	152	227	286	256	337
3	89	151	212	270	329	312
4	90	147	221	277	341	325
5	91	157	217	279	333	310
6	82	144	222	281	347	331
7	90	161	237	296	359	346
8	89	154	220	286	352	325
9	87	152	219	280	330	309
10	81	141	215	271	336	325
11						
12						

低剂量组

周	0w	1w	2w	3w	4w	空腹
日期	10.12	10.19	10.26	11.2	11.10	11.11
1	82	134	192	254	300	293
2	92	155	218	275	335	315
3	89	152	218	276	326	315
4	80	137	198	252	296	288
5	88	155	229	290	349	335
6	82	138	210	258	323	307
7	86	150	211	269	317	309
8	92	164	243	297	362	350
9	93	166	242	313	388	369
10	89	156	227	291	354	331
11						
12						

中剂量组

周	0w	1w	2w	3w	4w	空腹
1	86	143	213	262	344	312
2	85	149	218	260	337	316
3	88	150	223	287	342	335
4	83	141	202	260	314	306
5	89	148	216	267	321	310
6	90	155	218	269	340	325
7	86	148	222	287	354	342
8	90	142	208	260	322	317
9	82	135	202	264	324	308
10	88	154	220	280	344	322
11						
12						

高剂量组

周	0w	1w	2w	3w	4w	空腹
1	88	156	219	287	336	316
2	92	151	212	257	325	308
3	88	158	219	280	340	326
4	80	132	201	267	335	318
5	79	139	198	259	318	310
6	86	145	225	263	323	303
7	82	132	186	252	313	294
8	89	144	198	274	329	323
9	90	143	208	255	305	296
10	84	143	214	265	333	311
11						
12						

检验人/记录人：吴俊　　　　审核人：[illegible]　　　　日期：2017 2.23
