# Supplementary material for: Safety evaluation of Aloe vera soft capsule in acute, subacute toxicity and genotoxicity study
Source: PLoS One. 2021 Mar 26;16(3):e0249356. doi: 10.1371/journal.pone.0249356 (PMC7997006; doi:10.1371/journal.pone.0249356)
Supplement: S6 File — (PDF) [file pone.0249356.s006.pdf]

30天喂养试验大鼠解剖脏器重量原始记录（g）

样 品 编 号：G20160028	解剖房间号：901
实 验 日 期：2016.11.11　♀	环境温度：21 ℃
脏器秤编号：05-702	相对湿度：68 %

对照组					低剂量组				
	肝	肾	脾	睾丸		肝	肾	脾	睾丸
1	8.32	1.74	0.47		1	7.53	1.44	0.46	
2	9.01	1.95	0.59		2	8.4	1.7	0.52	
3	8.29	1.93	0.45		3	7.77	1.56	0.57	
4	8.7	1.88	0.5		4	7.64	1.61	0.51	
5	7.27	1.8	0.47		5	7.21	1.52	0.48	
6	8.17	1.92	0.53		6	7.69	1.76	0.53	
7	6.56	1.46	0.46		7	7.28	1.69	0.5	
8	7.66	1.73	0.56		8	9.29	1.87	0.68	
9	8.34	1.65	0.53		9	7.17	1.65	0.49	
10	6.98	1.83	0.39		10	9.8	1.94	0.51	
11					11				
12					12				

中剂量组					高剂量组				
	肝	肾	脾	睾丸		肝	肾	脾	睾丸
1	6.55	1.68	0.41		1	7.1	1.53	0.42	
2	6.66	1.48	0.59		2	7.81	1.86	0.6	
3	8.29	1.82	0.57		3	6.33	1.5	0.43	
4	7.49	1.7	0.54		4	7.26	1.71	0.45	
5	7.92	1.55	0.49		5	6.32	1.64	0.38	
6	7.59	1.72	0.43		6	6.27	1.48	0.36	
7	8.85	2.07	0.59		7	5.61	1.54	0.42	
8	6.54	1.9	0.64		8	9.35	1.67	0.58	
9	8.68	1.49	0.41		9	6.63	1.77	0.36	
10	6.47	1.43	0.38		10	8.86	1.94	0.54	
11					11				
12					12				

解剖所见异常			
编 号	肉眼见异常描述	编 号	肉眼见异常描述

检验/记录人：吴俊　　　　审核人：[illegible]　　　　审核日期：2017.2.23.

30天喂养试验大鼠解剖脏器重量原始记录（g）

样品编号：G202020160028		解剖房间号：901	
实验日期：2016.11.11　♂		环境温度：21 ℃	
脏器秤编号：05- 702		相对湿度：68 %	

对照组					低剂量组				
	肝	肾	脾	睾丸		肝	肾	脾	睾丸
1	10.1	2.42	0.79	2.97	1	9.47	2.23	0.65	3.1
2	12.32	2.89	0.69	3.15	2	11.06	3.01	0.71	3.23
3	10.18	2.4	0.69	2.91	3	11.55	2.81	0.66	3.39
4	11.26	2.7	0.7	3.21	4	10.02	2.45	0.73	3.21
5	10.19	2.29	0.78	3.37	5	11.37	2.76	1.06	3.16
6	10.92	2.57	0.82	3.29	6	11.16	2.5	0.84	3.36
7	12.04	2.57	0.94	3.41	7	10.24	2.41	0.88	2.98
8	11.99	3.04	0.83	3.05	8	13.11	2.71	0.66	3.4
9	10.56	2.13	0.71	2.75	9	13.84	2.7	1.02	3.38
10	11.93	2.44	0.7	3.22	10	10.69	2.46	0.83	3.52
11					11				
12					12				

中剂量组					高剂量组				
	肝	肾	脾	睾丸		肝	肾	脾	睾丸
1	11.48	2.19	0.76	3.12	1	11.88	2.8	0.85	3.15
2	10.82	2.73	1.06	3.16	2	11.09	2.34	0.73	3.71
3	11.66	2.61	0.89	3.21	3	10.42	2.22	0.77	3.39
4	10.98	2.47	0.71	3.3	4	10.04	2.28	0.95	3.16
5	10.67	2.5	0.81	3.28	5	10.79	2.43	0.92	3.13
6	11.11	2.78	0.76	3.46	6	10.08	2.37	0.72	3.24
7	12.6	3	0.85	3.58	7	9.41	2.34	0.64	3.18
8	11.76	2.22	0.96	2.99	8	12.09	2.63	0.8	3.18
9	10.97	2.6	0.8	3.16	9	9.22	2.58	0.82	3.05
10	11.81	2.52	0.81	3.3	10	9.96	2.19	0.67	2.89
11					11				
12					12				

解剖所见异常			
编 号	肉眼见异常描述	编 号	肉眼见异常描述

检验/记录人：吴俊　　　审核人：[illegible]　　　审核日期：2017.2.23.
